# Assessing the reliability of predicted plant trait distributions at the global scale

**DOI:** 10.1111/geb.13086

**Published:** 2020-03-20

**Authors:** Coline C. F. Boonman, Ana Benítez‐López, Aafke M. Schipper, Wilfried Thuiller, Madhur Anand, Bruno E. L. Cerabolini, Johannes H. C. Cornelissen, Andres Gonzalez‐Melo, Wesley N. Hattingh, Pedro Higuchi, Daniel C. Laughlin, Vladimir G. Onipchenko, Josep Peñuelas, Lourens Poorter, Nadejda A. Soudzilovskaia, Mark A. J. Huijbregts, Luca Santini

**Affiliations:** ^1^ Department of Environmental Science Institute for Water and Wetland Research Radboud University Nijmegen the Netherlands; ^2^ Integrative Ecology Group Estación Biológica de Doñana (EBD‐CSIC) Sevilla Spain; ^3^ PBL Netherlands Environmental Assessment Agency The Hague the Netherlands; ^4^ Université Grenoble Alpes, CNRS, University of Savoie Mont Blanc LECA, Laboratoire d’Écologie Alpine Grenoble France; ^5^ School of Environmental Sciences University of Guelph Guelph Ontario Canada; ^6^ Department of Theoretical and Applied Science University of Insubria Varese Italy; ^7^ Systems Ecology Department of Ecological Science Vrije Universiteit Amsterdam the Netherlands; ^8^ Facultad de Ciencias Naturales y Matemáticas Universidad del Rosario Bogota Colombia; ^9^ School of Animal, Plant and Environmental Sciences University of the Witwatersrand Johannesburg South Africa; ^10^ Forestry Department Santa Catarina State University Lages Brazil; ^11^ Department of Botany University of Wyoming Laramie WY USA; ^12^ Department of Geobotany Moscow Lomonosov State University Moscow Russia; ^13^ CREAF, Vallès Catalonia Spain; ^14^ CSIC, Global Ecology Unit CREAF‐CEAB‐UAB Catalonia Spain; ^15^ Forest Ecology and Forest Management Group Wageningen University and Research Wageningen the Netherlands; ^16^ Environmental Biology Department Institute of Environmental Sciences Leiden University Leiden the Netherlands; ^17^ National Research Council Institute of Research on Terrestrial Ecosystems (CNR‐IRET) Monterotondo Italy

**Keywords:** ensemble forecasting, environmental filtering, intraspecific trait variation, leaf nitrogen concentration, plant height, specific leaf area, trait–environment relationships, trait model, wood density

## Abstract

**Aim:**

Predictions of plant traits over space and time are increasingly used to improve our understanding of plant community responses to global environmental change. A necessary step forward is to assess the reliability of global trait predictions. In this study, we predict community mean plant traits at the global scale and present a systematic evaluation of their reliability in terms of the accuracy of the models, ecological realism and various sources of uncertainty.

**Location:**

Global.

**Time period:**

Present.

**Major taxa studied:**

Vascular plants.

**Methods:**

We predicted global distributions of community mean specific leaf area, leaf nitrogen concentration, plant height and wood density with an ensemble modelling approach based on georeferenced, locally measured trait data representative of the plant community. We assessed the predictive performance of the models, the plausibility of predicted trait combinations, the influence of data quality, and the uncertainty across geographical space attributed to spatial extrapolation and diverging model predictions.

**Results:**

Ensemble predictions of community mean plant height, specific leaf area and wood density resulted in ecologically plausible trait–environment relationships and trait–trait combinations. Leaf nitrogen concentration, however, could not be predicted reliably. The ensemble approach was better at predicting community trait means than any of the individual modelling techniques, which varied greatly in predictive performance and led to divergent predictions, mostly in African deserts and the Arctic, where predictions were also extrapolated. High data quality (i.e., including intraspecific variability and a representative species sample) increased model performance by 28%.

**Main conclusions:**

Plant community traits can be predicted reliably at the global scale when using an ensemble approach and high‐quality data for traits that mostly respond to large‐scale environmental factors. We recommend applying ensemble forecasting to account for model uncertainty, using representative trait data, and more routinely assessing the reliability of trait predictions.

## 1 INTRODUCTION

Global trait‐based models have proliferated in recent years owing to the increasing availability of plant trait data (Kattge, 2019). Fitting and projecting trait–environment relationships over large spatial scales is becoming increasingly common to study trait trade‐offs (e.g., Díaz et al., 2016; Wright et al., 2004), to relate traits to environmental gradients (e.g., Moles et al., 2009; Wright et al., 2005) and to describe geographical patterns of traits (e.g., Madani et al., 2018; Yang et al., 2016). Trait‐based models can not only increase our understanding of trait–environment relationships, but can also allow us to estimate how plant traits might respond to global environmental change (Bjorkman et al., 2018). This, in turn, is considered particularly useful for predicting the impact of environmental change on vegetation (Webb, Hoeting, Ames, Pyne, & LeRoy Poff, 2010), because trait‐based models allow to directly link plant fitness to environmental filters, including climate, soil properties and disturbance (Keddy, 1992).

Recent attempts to model global plant trait distributions as a function of environmental conditions have yielded different trait patterns (e.g., Butler et al., 2017; Madani et al., 2018; Moreno‐Martínez et al., 2018; Van Bodegom, Douma, & Verheijen, 2014). For example, Butler et al. (2017) and Moreno‐Martínez et al. (2018) predicted specific leaf area to be low in western Canada and high in northern Russia and eastern Brazil, whereas Van Bodegom et al. (2014) and Madani et al. (2018) predicted the opposite. Such discrepancies among existing global trait models might arise from differences in: (a) the sources of the underlying trait data (floras and measurements in natural areas, manipulated field experiments, botanical gardens and greenhouses); (b) the representativeness of the sampled species for the entire natural plant community; (c) the selection of environmental predictors; and (d) the model fitting techniques. The first difference translates to the use of global species trait averages, which may cause a potential mismatch of trait and environmental data. The second and third differences may result in different spatial patterns of uncertainty owing to extrapolations of trait–environment relationships (Thuiller, Brotons, Araujo, & Lavorel, 2004). The fourth difference may render considerable variation among predictions (Thuiller, Guéguen, Renaud, Karger, & Zimmerman, 2019). Furthermore, the ecological realism of the combination of predicted plant traits needs to be tested against observed trait combinations, which has, to our knowledge, not yet been done at the global scale. Additionally, although all studies reported the variance explained by the trait‐based models, the predictability of independent samples (i.e., data not used to train the models) has not yet been assessed thoroughly. A necessary step forward to increase macroecological insights in global trait–environment relationships with potential application in ecological impact or conservation assessments (e.g., Lavorel & Garnier, 2002; Madani et al., 2018) is to perform a thorough assessment of the reliability of global plant trait predictions.

Here, we predict community mean plant traits at the global scale and present a systematic evaluation of the reliability of the predictions in terms of the models' accuracy, ecological realism and various sources of uncertainty. We systematically selected locally measured, representative data focusing on four widely studied plant traits (specific leaf area, leaf nitrogen concentration, height and wood density). These traits reflect the global spectra in plant form and function, are responsive to the abiotic environment and show physical trade‐offs with other traits (Table 1; Díaz et al., 2016; Lavorel & Garnier, 2002). For each of the traits, we calculated community mean trait values and predicted global patterns at a 0.5° resolution (*c*. 55 km × 55 km at the equator) using an ensemble modelling approach based on two regression and two machine learning techniques. Subsequently, we evaluated the predictive performance of the models and assessed their ecological plausibility in terms of the trait–environment relationships and the correlations and combinations of individually predicted community mean trait values (Díaz et al., 2016; Lavorel & Garnier, 2002). Finally, we evaluated the effect of various sources of uncertainty: (a) the effect of data quality in terms of representativeness of the sampled species to the entire plant community and the use of global species trait averages versus local trait measurements; (b) the uncertainty across geographical space attributed to extrapolation of traits outside the applicability domain (i.e., the geographical area with environmental variation covered by the environmental variation of the trait data); and (c) the uncertainty across geographical space owing to discrepancies among the predictions of the four modelling techniques. We build upon this assessment to provide guidelines for the further development, interpretability and usability of global trait‐based models.

**TABLE 1:** Expectations for community mean trait–environment relationships

Predictor	Rationale	Impact and adaptations	Expectations
Minimum temperature of the coldest month (Tmin)	Temperature‐induced leaf damage, at low and high Tmin, affects plant performance^1^	Lower damage risk with increased tolerance via decreasing SLA^2^	˄ SLA
		Enhanced metabolic activity and frost tolerance with increased LNC^1,3^	↓ LNC^4^
	Lower frost‐induced mortality risk with increasing Tmin		↑ Plant height^4^
			↑ Wood density^4^
Humidity index (HumInd)	Lower drought‐induced mortality risk with increasing HumInd	Increased drought tolerance with low transpiration rates via decreased SLA	↑ SLA^2^
			↑ LNC^2^
		Cavitation risk increases with height^5^	↑ Plant height
		Increasing wood density increases plant performance at low HumInd^6^	↓ Wood density
Precipitation in the driest quarter of the year (PrecDryQ)	Lower drought‐induced mortality risk with increasing PrecDryQ	Cavitation risk increases with height^5^	ᴑ SLA
			ᴑ LNC
			↑ Plant height
			ᴑ Wood density
Precipitation seasonality (PrecSeas)	Moisture availability affects plant survival	Changing leaf habit (deciduousness) not traits^7^	‒ SLA
			‒ LNC^4^
			↓ Plant height^4^
			↓ Wood density^4^
Soil cation exchange capacity (CEC)	Higher CEC indicates a greater capacity to retain easily attainable nutrients (i.e., higher soil fertility)^8^	Less durable structures can be maintained^9,10^	↑ SLA
			‒ LNC
			ᴑ Plant height
			↓ Wood density
Soil pH	Higher pH increases the available phosphorus and nitrogen in the soil (i.e., higher soil fertility)^11^	Less durable structures can be maintained^9,10^	↑ SLA
			‒ LNC
			ᴑ Plant height
			↓ Wood density

Note

Hypotheses are indicated as follows: ↑ = positive relationship; ↓ = negative relationship; ‒ = flat relationship; **˄ = **unimodal response. Hypotheses are based on theory (no reference) or on significant, biologically relevant trends found at the community level in literature (reference included). ᴑ indicates relationships between trait and predictor that have not been discussed at the community level in literature.

Abbreviations: LNC = leaf nitrogen concentration; SLA = specific leaf area.

References in table: ^1^Went (1953); ^2^Wright et al. (2005); ^3^Reich, Oleksyn, and Tjoelker (1996); ^4^Swenson and Weiser (2010); ^5^Tyree and Sperry (1989); ^6^Reich (2014); ^7^Borchert (1998); ^8^Ross and Ketterings (1995); ^9^Chave et al. (2009); ^10^Wright et al. (2004); ^11^Maire et al. (2015).

## 2 METHODS

### 2.1 Plant functional traits

We selected four plant functional traits: specific leaf area (SLA; in square millimetres per milligram), leaf nitrogen concentration (LNC; in milligrams per gram), height (in metres) and wood density (in milligrams per cubic millimetre). The SLA and LNC are both linked to photosynthetic capacity and nutrient investment, where a high SLA and high LNC represent a fast return on investment at the expense of a shorter life span (Wright et al., 2004). Plant height is considered to be indicative of the ecological strategy of carbon distribution, indirectly determining growth and reproduction and their initial response to climate change (Moles et al., 2009). Last, wood density is a measure of carbon investment, representing a trade‐off between growth (e.g., to overcome light limitation) and strength (e.g., mechanical support and drought tolerance), with higher wood density reflecting slower growth but increased strength at a similar stem diameter (Chave et al., 2009; Larjavaara & Muller‐Landau, 2010).

The selected traits are easily measured according to standardized measurement procedures (Perez‐Harguindeguy et al., 2013), which facilitates the integration of data from multiple datasets and reduces trait variation caused by seasonal changes (Bloomfield et al., 2018). To optimize between the number of observations and the consistency of the measurement methods, we included SLA measurements on both sun‐leaves and shade‐leaves, wood density measurements on both heartwood and sapwood, and plant height measurements on both vegetative and generative plant organs (see also Siefert et al., 2015).

### 2.2 Data collection and selection procedure

Our main source for plant trait data was the TRY database (Kattge et al., 2011), from which we received 964,464 trait records on 85,437 species from 168 datasets. We also obtained data from the Tundra Trait Team (TTT) database (Bjorkman et al., 2018) and from various other published and unpublished datasets (Supporting Information Appendix S1). A list of data sources is provided in the Appendix. All species names were standardized using The Plant List (2013).

We selected trait observations based on six criteria. First, we included only georeferenced observations in order to enable a meaningful link with environmental covariates. Second, we considered only real measurements of plant traits (i.e., no species‐level averages) in order to include intraspecific trait variation, which can contribute substantially to the trait variation within and between communities (Albert et al., 2010; Bloomfield et al., 2018; Siefert et al., 2015). Third, we considered only measurements obtained from natural vegetation, to minimize the influences of local management practices and legacy effects of historical land use (Perring et al., 2017). Fourth, we included observations only from studies that measured all or the most abundant species present in the entire plant community or in the dominant vegetation structure, in order to account for the representativeness of the sampled species for the plant community. This is in line with previous studies on this topic (e.g., Poorter et al., 2017) and in accordance with the biomass ratio hypothesis (Grime, 1998). Fifth, we included observations only from studies that targeted all life stages and/or size classes and from studies that targeted only adults. Thus, we excluded observations from studies focusing only on early‐successional plant communities and studies measuring only seedlings or juveniles in a more established vegetation, in order to reduce confounding effects of ontogeny (e.g., Thomas & Bazzaz, 1999) and succession (e.g., Purschke et al., 2013). Sixth, we included only measurements conducted from 1980 onwards, in order to link up with the environmental covariate data, which span from 1979 to 2013. We excluded trait observations for which the listed criteria could not be checked. The Supporting Information (Appendix S2) contains an overview of the number of datasets excluded per criterion. In total, we removed 63.1% of all the datasets we originally received from TRY and 54.5% of all datasets in the TTT database.

### 2.3 Data processing

We checked and corrected for possible errors in our database, such as duplicates, coordinate and unit inaccuracies and outliers (see Supporting Information Appendix S2). We then calculated location‐specific community means per trait and per study to represent the mean response of the plant community to the local environment (Ackerly & Cornwell, 2007). Using unweighted community means was the better compromise over abundance‐weighted community means, mainly because previous studies were not conclusive on the superiority of abundance‐weighted means and because > 50% of our data did not include species abundances (for further discussion, see Supporting Information Appendix S3). We averaged the location‐specific community means to 0.5° grid cells (with a median of 22 communities per grid cell) in order to describe large‐scale patterns in traits. This is a common resolution used in global plant trait studies (Butler et al., 2017; Van Bodegom et al., 2014) and a good compromise, accounting for the effect of climatic filtering on plant traits, the coordinate uncertainty of the trait data, avoidance of pseudo‐replicates and the uncertainty related to interpolated climatic variables (Stoklosa, Daly, Foster, Ashcroft, & Warton, 2015). These community means represent plant community average trait values, thereby making values not directly transferable to a specific species or a class of species. The final dataset used as input for model fitting included community mean trait values based on data from 76 studies and 8,955 species averaged to 486 grid cells, including 361 grid cells for SLA, 338 for LNC, 217 for height and 125 for wood density (Supporting Information Figure S4).

### 2.4 Environmental data

We considered environmental variables that are expected to affect plant performance (e.g., Kimball, Gremer, Angert, Huxman, & Venable, 2012). This allowed us to check the ecological plausibility of the resulting trait–environment relationships. Based on ecological relevance, we selected bioclimatic variables from CHELSA v.1.2 (Karger et al., 2017). Given that water availability to plants is not determined by precipitation alone, we calculated the aridity index as the mean annual precipitation divided by the mean annual potential evapotranspiration, which in turn was calculated using the Penman–Monteith model (Zomer, Trabucco, Bossio, & Verchot, 2008). However, given that lower values of the aridity index indicate higher aridity, to avoid confusion this predictor will be referred to as the “humidity index” from now on. Furthermore, we selected soil characteristics from SoilGrids250m (Hengl et al., 2017) and resampled them to a resolution of 0.5° to match the resolution of the plant community mean trait data. We averaged the soil data to a depth of 30 cm, which we considered most relevant for community composition via plant establishment and by influencing plants in later life stages, for example, through the potentially high nutrient availability (e.g., Vitousek & Sanford, 1986).

To avoid collinearity, we reduced the number of predictors based on variance inflation factors (VIF < 4), while ensuring a combination of different environmental factors (i.e., both climatic and soil characteristics; Franklin, Serra‐Diaz, Syphard, & Regan, 2016). A complete list of the predictors considered and more details on our selection procedure can be found in the Supporting Information (Appendix S5). The final selection included six predictors: minimum temperature of the coldest month (Tmin; in degrees Celsius), humidity index (HumInd; dimensionless), precipitation in the driest quarter of the year (PrecDryQ; in millimetres), precipitation seasonality (PrecSeas, coefficient of variation based on monthly precipitation; as a percentage), soil cation exchange capacity (CEC; centimoles of positive charge per kilogram of soil) and soil pH.

### 2.5 Model fitting and validation

For each relationship between the plant traits and environmental variables, we formulated expectations on the shape of the response based on existing literature (Table 1). To quantify the trait–environment relationships, we fitted four different models per trait: two statistical models [generalized linear model (GLM) and generalized additive model (GAM)] and two machine learning models [random forest (RF) and boosted regression trees (BRT); for full details on model parameterization, see Supporting Information Appendix S6]. We validated all models by running a 10‐fold cross‐validation using a split‐sample procedure (80%–20%) and evaluated the predictive performance (cross‐validated pseudo‐*R* ^2^) of the models by regressing the predicted and observed trait values from all repetitions of the cross‐validation.

To quantify the relative importance of each predictor in a consistent way across the models, we predicted traits using permuted values for the predictor of concern, correlated those predictions with predictions of the model using the original data and quantified relative variable importance as one minus the Spearman rank correlation coefficient (Thuiller, Lafourcade, Engler, & Araújo, 2009).

### 2.6 Testing sensitivity to data quality

To test the influence of data quality on the performance of the trait models, we created three alternative datasets differing in terms of trait values (i.e., including intraspecific trait variation versus using species‐specific trait values) and in terms of species representativeness (i.e., a representative sample of species versus the random selection of one species in the plant community; Supporting Information, Figure S7). For each alternative dataset, we fitted the four different models for each of the four traits, following the procedure described above for our default dataset. We then evaluated each model separately, where the predictions are confronted with the observed community means of the full dataset (Supporting Information Appendix S7).

### 2.7 Spatial predictions and their assessment

To derive spatial predictions per trait, we used an ensemble forecasting procedure, which averages the predictions of the four algorithms weighted by their cross‐validated pseudo‐*R* ^2^ values (Marmion, Parviainen, Luoto, Heikkinen, & Thuiller, 2009). Likewise, we estimated ensemble variable importance and partial response curves. Furthermore, we calculated the accuracy of the ensemble predictions by regressing the 20% test data of the cross‐validations against the observed trait values. We checked whether among‐trait correlations observed in the data were retained in the predictions by correlating the trait correlation matrix of the original dataset with the trait correlation matrix of the predictions. Additionally, we assessed whether predicted trait combinations were realistic by comparing them with a hypervolume of all trait combinations existing in our dataset. We built the hypervolume with the “hypervolume_svm” function of the “hypervolume” R package using the original trait values before calculating community means or grid cell averages, because we wanted to check whether predicted trait combinations occurred naturally and because this ensured enough observations with all four traits measured to build the hypervolume (Blonder, Lamanna, Violle, & Enquist, 2014). Note that this test does not necessarily imply that predicted trait combinations do not or cannot exist, but merely that they did not occur in the input data and should therefore be interpreted with caution.

To identify the applicability domain, we calculated and mapped the multivariate environmental similarity surface (Elith, Kearney, & Phillips, 2010). This analysis quantifies, per grid cell, the difference of the most extrapolated environmental predictor and the environmental range of that predictor covered by locations in the plant trait dataset, while considering the distribution of these data within the global environmental range. Finally, to quantify model uncertainty (i.e., the variability in predictions across models), the coefficient of variation was calculated as the difference in the predictions of each of the four individual models compared with the ensemble prediction weighted by the predictive performance of each of the models (further explanation in Supporting Information Appendix S8).

## 3 RESULTS

### 3.1 Predicted global plant trait variation

**FIGURE 1:**
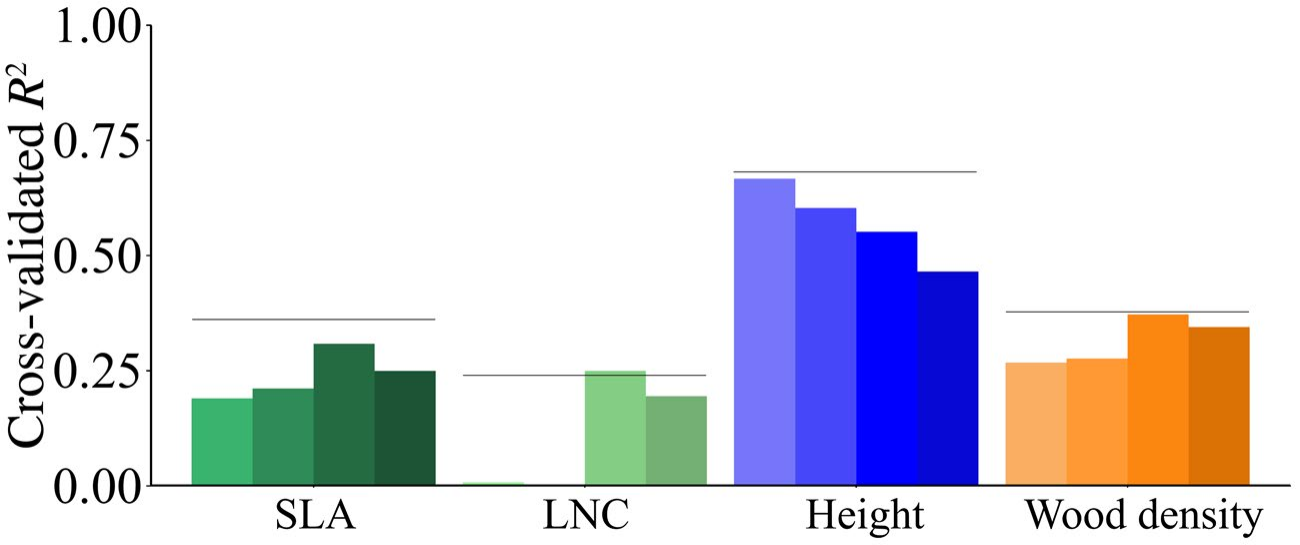
Model performance for different traits and different models presented as the cross‐validated *R* ^2^ values. Per trait, the bars represent the predictive performance of GLM, GAM, RF, and BRT from left to right. The horizontal black lines represent the accuracy of the ensemble predictions. Abbreviations: LNC = leaf nitrogen concentration; SLA = specific leaf area [Colour figure can be viewed at wileyonlinelibrary.com]

**FIGURE 2:**
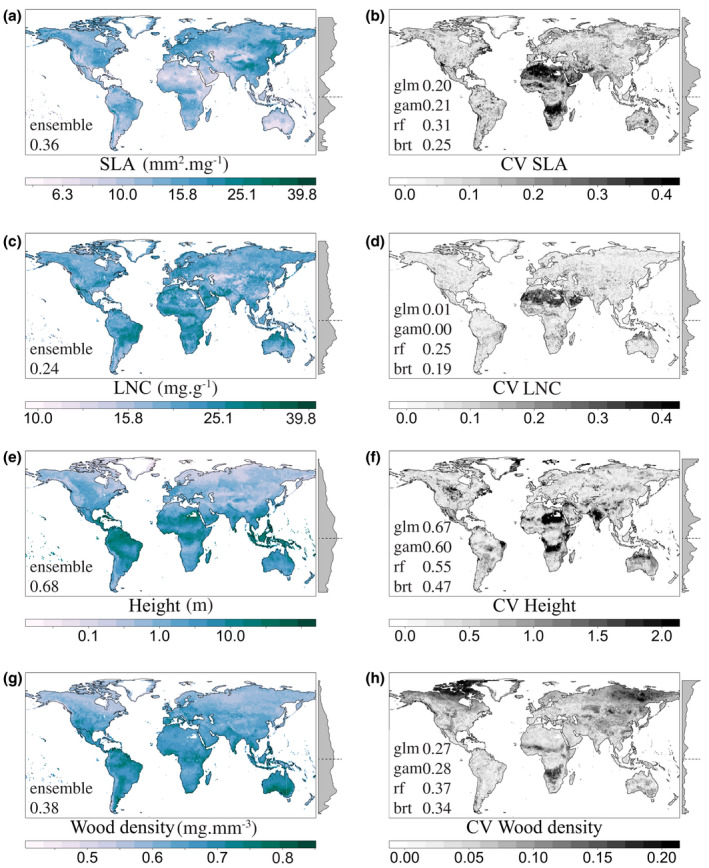
Global plant trait predictions. The left column presents the ensemble trait predictions, and the right column presents the ensemble coefficient of variation (CV) of different model predictions. The grey margin in each plot depicts the mean latitudinal value, and the horizontal dashed line indicates the Equator. Values in plots (a), (c), (e), (g) indicate the accuracy of the ensemble predictions (cross‐validated *R* ^2^). Values in plots (b), (d), (f), (h) indicate the predictive performance (cross‐validated *R* ^2^) of the different models, where glm indicates values of the GLMs, gam of GAMs, rf of RFs, and brt of BRT. These values indicate how well traits are predicted, while the CV maps only identify locations where predictions are divergent between models with high and/or similar predictive power. For extrapolation areas, see Figure 6. No predictions were made for Greenland (no soil data) , nor for the south of Egypt (no precipitation seasonality). All predictions represent potential trait values, as not all terrestrial world is vegetated or includes woody vegetation. Abbreviations: LNC = leaf nitrogen concentration; SLA = specific leaf area [Correction added on 16 April 2020, after first online publication: Figure 2 was previously incorrect and has been updated in this version.] [Colour figure can be viewed at wileyonlinelibrary.com]

The variation explained by the environmental variables varied among traits (Figure 1). Model predictive performance was highest for plant height, followed by wood density, SLA and LNC (Figure 1). The projected global trait maps revealed low SLA in arid areas and high SLA in temperate climates (Figure 2a), whereas LNC was found to be low in dry tropical regions (Figure 2c). We also found lower community plant height at higher latitudes, with the tallest vegetation in wet tropical areas and shorter vegetation in temperate climates (Figure 2e). Height values, like the other predicted traits, represent community means and thus cannot be transferred directly to a specific species or a class of species. Finally, wood density, representing the potential average wood density for all woody vegetation in a location, was predicted to be particularly low in areas with low PrecSeas (Figure 2g).

### 3.2 Ecological assessment

#### 3.2.1 Trait–environment relationships

**FIGURE 3:**
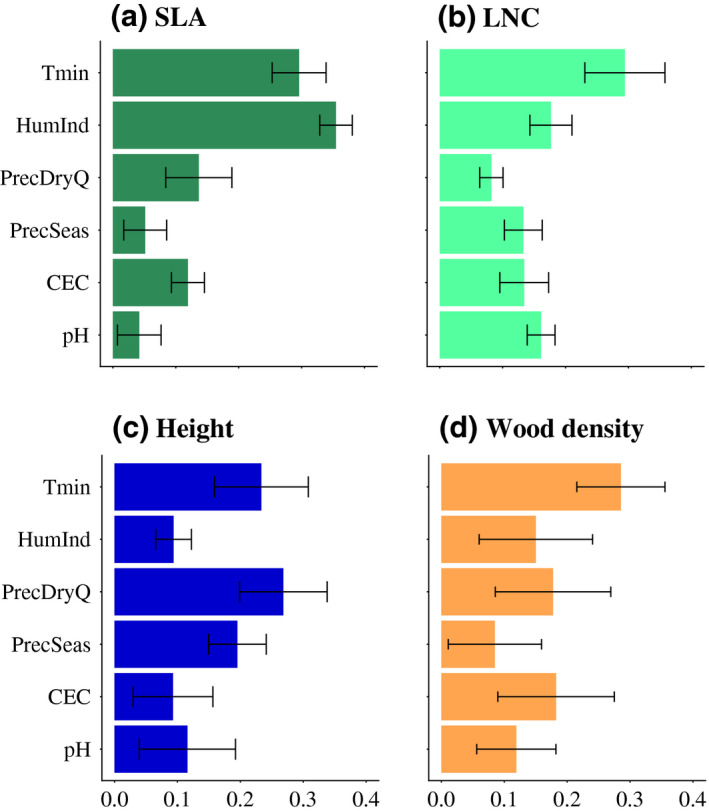
Relative predictor importance, calculated as 1—Spearman rank correlation coefficient (see Section 2.5), where high values indicate high importance. The bars represent the ensemble relative predictor importance and the error bars show the ensemble coefficient of variation. Abbreviations: LNC = leaf nitrogen concentration; SLA = specific leaf area; Tmin = minimum temperature; HumInd = humidity index; PrecDryQ = precipitation in the driest quarter of the year; PrecSeas = precipitation seasonality and CEC for soil cation exchange capacity [Colour figure can be viewed at wileyonlinelibrary.com]

**FIGURE 4:**
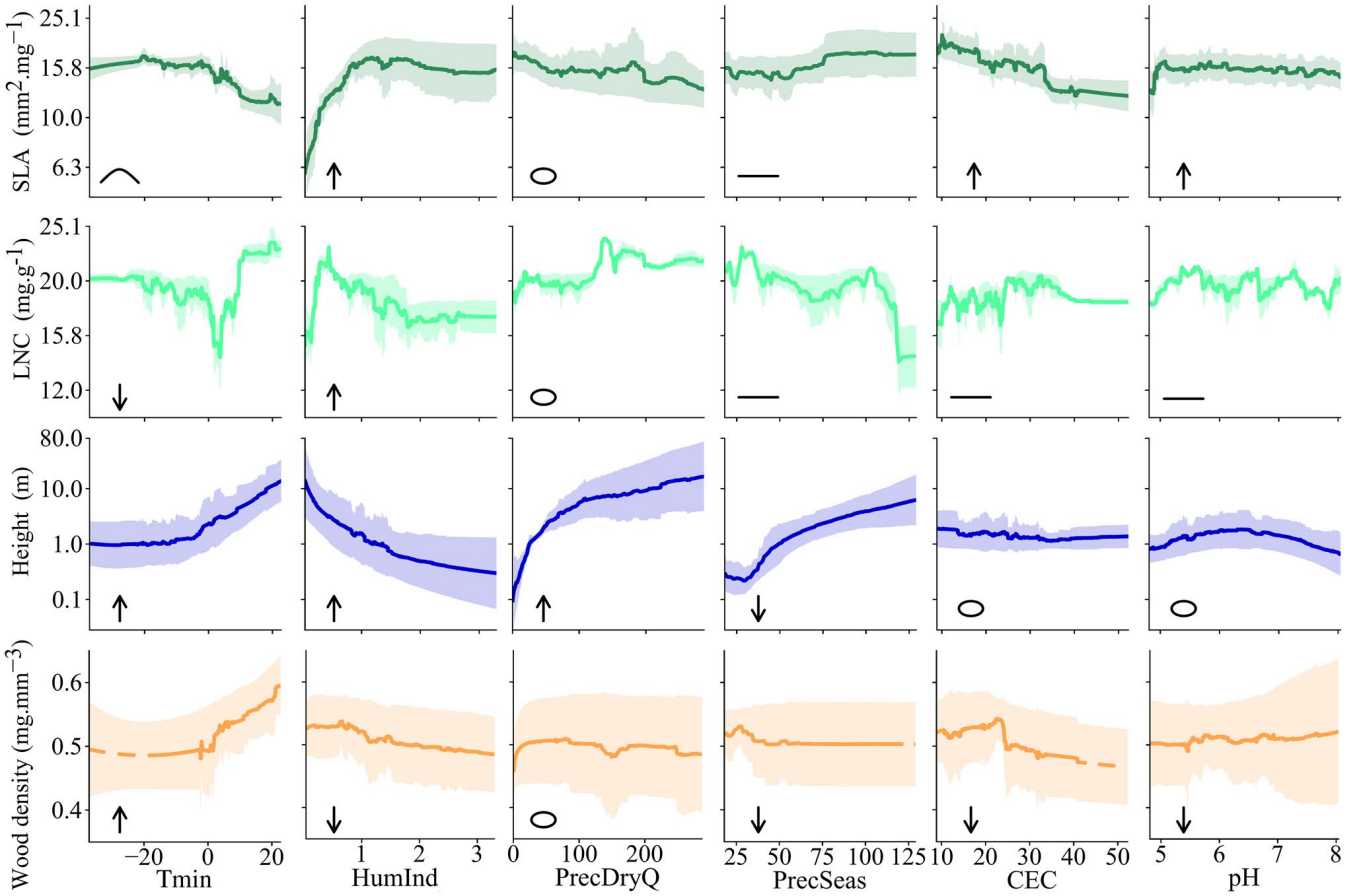
Ensemble partial trait responses. Shaded areas are the ensemble coefficients of variation. Predictors are minimum temperature (Tmin; °C), humidity index (HumInd; unitless), precipitation in the driest quarter of the year (PrecDryQ; mm), precipitation seasonality (PrecSeas; %), soil cation exchange capacity (CEC; cmol+ kg^‐1^), and soil pH. Extrapolated trait responses are shown with a dashed line. The symbols at the bottom left of each panel represent the expectation reported in Table 1: ↑ = positive relationship; ↓ = negative relationship; ‒ = flat relationship; **˄** = unimodal response; ᴑ = the relationship has not been discussed at the community level in literature. Other Abbreviations: LNC = leaf nitrogen concentration; SLA = specific leaf area [Colour figure can be viewed at wileyonlinelibrary.com]

In our models, community mean SLA was mostly explained by HumInd and Tmin (Figure 3a). As expected, SLA showed a unimodal albeit mostly decreasing response to Tmin, an increase with HumInd and a flat response to PrecSeas. However, we did not find the expected response of SLA to soil CEC and soil pH (Table 1; Figure 4). We also found a flat response of SLA to PrecDryQ (Figure 4). Community mean LNC was mostly explained by Tmin (Figure 3b). We expected LNC to decrease with Tmin, to increase with HumInd and to show a flat response to PrecSeas, but we found a unimodal response of LNC with Tmin around 0 °C, and a decrease of LNC with HumInd and PrecSeas (Table 1; Figure 4). We also found LNC to increase with PrecDryQ and, as expected, to show a flat response to soil CEC and soil pH (Table 1; Figure 4). Community mean plant height was mostly explained by PrecDryQ (Figure 3c). As expected, height increased with increasing Tmin and PrecDryQ, whereas in contrast to our original expectation, we found height to decrease with HumInd and to increase with PrecSeas (Table 1; Figure 4). Height showed a flat response to soil CEC and a unimodal response to soil pH. Community mean wood density was mostly explained by Tmin (Figure 3d). As expected, wood density increased with Tmin, decreased with HumInd and decreased overall with soil CEC (Table 1; Figure 4). Against our expectations, wood density showed a flat response to PrecSeas and soil pH (Table 1; Figure 4). Furthermore, we found a flat response of wood density to PrecDryQ (Figure 4).

#### 3.2.2 Combinations of predicted traits

The predicted trait values largely preserved the global trait correlations present in the training data (*r* = .83). Furthermore, for 99.9% of all grid cells, the predicted trait combination of community mean traits was found within the existing trait combinations found in individual plants or species. Locations with unrealistic predicted trait combinations are presented in the Supporting Information (Figure S9).

### 3.3 Uncertainty assessment

#### 3.3.1 Data quality

**FIGURE 5:**
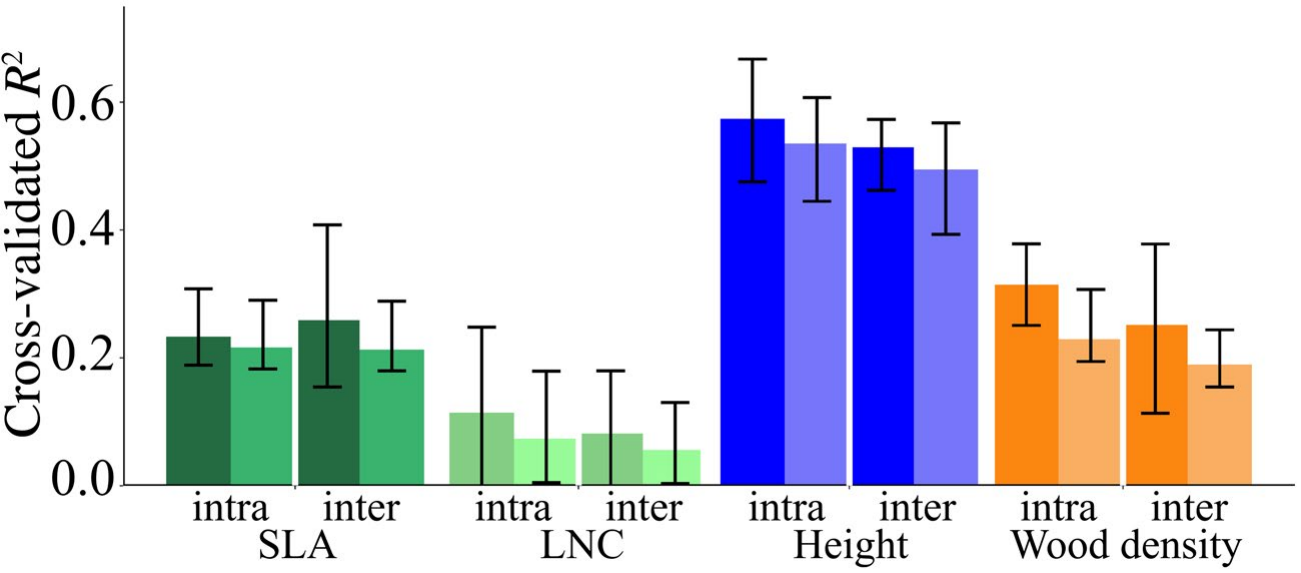
Performances of models based on datasets of different quality. The bars represent the average cross‐validated *R* ^2^ values over the four different models, where the errorbars indicate the minimum and maximum cross‐validated *R* ^2^ value. Datasets including and excluding intraspecific trait variation are indicated with intra and inter, respectively. Species representativeness is indicated with the shade of the bar, where darker shades represent datasets with a species sample representative of the community and lighter bars represent datasets with a non‐representative species sample (Appendix S7). Thus, per trait, the bars from left to right represent plot 1, 2, 3, and 4 of Appendix Figure S7. Abbreviations: LNC = leaf nitrogen concentration; SLA = specific leaf area [Colour figure can be viewed at wileyonlinelibrary.com]

The predictive accuracy decreased by 11% on average across all traits and models when intraspecific trait variation was excluded (Figure 5; Supporting Information Table S7). When species were sampled randomly (i.e., an unrepresentative sample), the predictive accuracy of the models decreased on average by 19% (Figure 5). The combination of ignoring intraspecific trait variation and using a non‐representative species sample amplified the reduction in accuracy to 28% compared with the default models (Figure 5).

#### 3.3.2 Applicability domain

**FIGURE 6:**
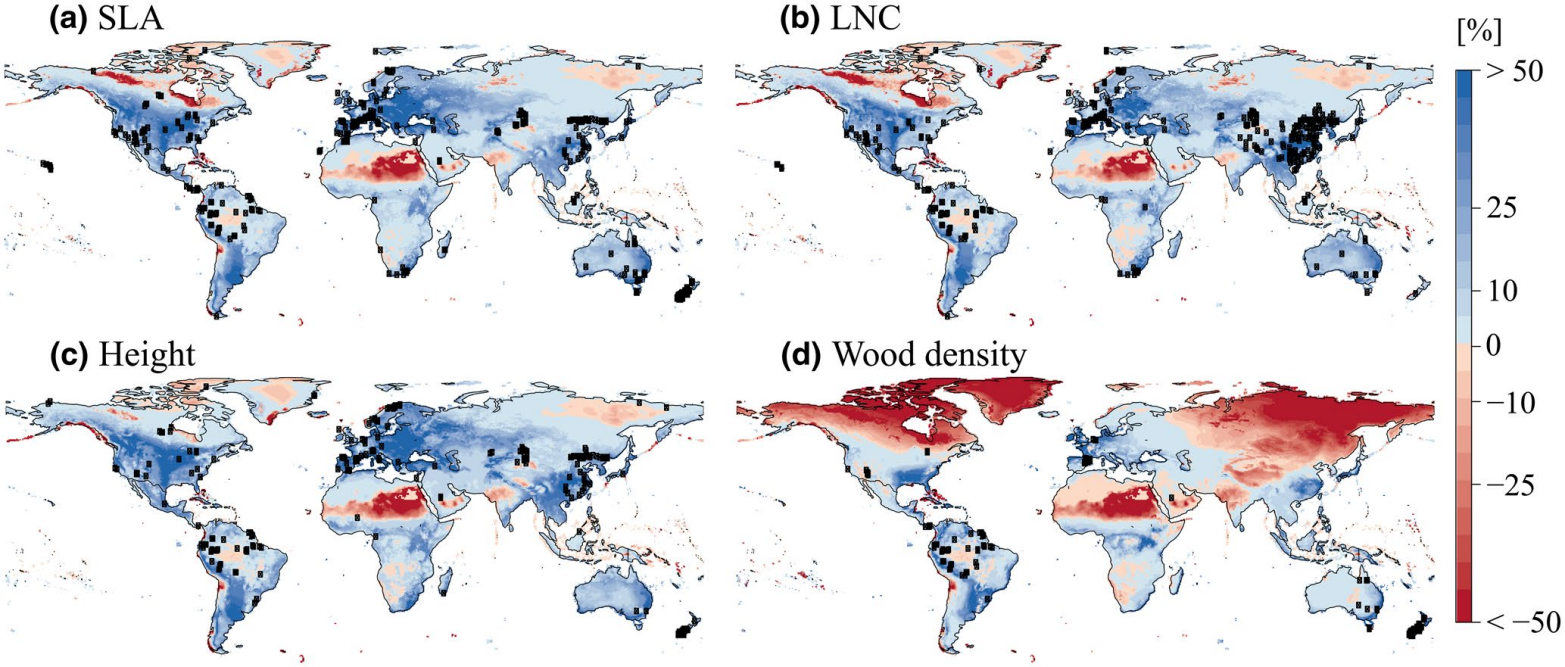
Locations of trait observations (black dots) and the environmental coverage of trait observations, that is, the multivariate environmental similarity surface (Elith et al., 2010), where blue represents interpolation and red represents extrapolation. More intense shades indicate greater similarities (blue) or differences (red) in environmental conditions of the location compared to the environmental conditions covered by the training dataset. Though values indicate percentages, each griddcell might be restrained by a different environmental variable, which is why the actual values are difficult to interpret. Abbreviations: LNC = leaf nitrogen concentration; SLA = specific leaf area [Colour figure can be viewed at wileyonlinelibrary.com]

Despite data paucity in large areas of the world (e.g., India, Asian Russia and Africa; Figure 6), the trait data covered a large part of the global environmental space (Figure 6; Supporting Information Figure S10). However, deserts, tropical islands and some parts of the Arctic were outside the environmental domain covered by the trait data.

#### 3.3.3 Model selection

Predictive performance differed between models per trait, where RF showed the highest predictive performance for SLA, LNC and wood density, and GLM for height (Figure 1). The ensemble predictions were always equal to or better than the individual models (Figure 1). The consistency in the predictions among the four modelling techniques varied geographically (Figure 2b,d,f,h). The coefficient of variation of SLA was mostly low, apart from some areas in Africa. LNC had an overall low coefficient of variation, except for the Sahara. Plant height showed a shifting pattern of high and low coefficients of variation over the globe. Wood density had an low coefficient of variation overall but was predicted with more uncertainty in parts of Africa and most of the Arctic.

## 4 DISCUSSION

In this study, we established global plant trait models based on locally measured trait data representative of natural vegetation and used these models to make global trait predictions. We evaluated these trait predictions based on the predictive performance of the models, their ecological realism, the influence of the quality of input data, the applicability domain of the predictions and model choice uncertainty. Our results showed that the models predicting community mean SLA, plant height and wood density were well able to explain trait variation. Although the trait–environment relationships were as expected only in part, our trait predictions preserved the observed among‐trait correlations and combinations. Furthermore, we showed that most of the global environmental variation was within the applicability domain for trait predictions, that including intraspecific trait variation and having a representative species sample improved the predictability of traits at the resolution and extent applied here, that different modelling techniques demonstrated different predictive performances for different traits and that the ensemble approach was better at predicting traits. Overall, these findings suggest that global predictions aimed at describing broad geographical plant community mean trait patterns are reliable with the data currently available, but caution is needed for certain traits and areas.

### 4.1 Global trait patterns

The variance explained by our models predicting global community mean trait values along environmental gradients is comparable with other global or large‐scale trait‐based studies (Butler et al., 2017; Madani et al., 2018; Van Bodegom et al., 2014; Yang et al., 2016). For some areas, predicted spatial patterns were consistent with those predicted by previous studies. For example, we found lower SLA values in central Australia compared with the coast (Figure 2a), demonstrating the adaptation of leaves to drier conditions (Butler et al., 2017; Madani et al., 2018; Moreno‐Martínez et al., 2018; Van Bodegom et al., 2014). Likewise, we found lower SLA values in the Amazon compared with the South American Cerrado (Figure 2a; Butler et al., 2017; Moreno‐Martínez et al., 2018). Furthermore, we predicted vegetation in tropical climates to be taller than in temperate climates (Figure 2; Madani et al., 2018).

On the contrary, our SLA, LNC and wood density predictions differed from the predictions of previous studies for some areas. For example, we predicted higher SLA values for boreal forests compared with desert areas, similar to Van Bodegom et al. (2014) but opposite to Butler et al. (2017). Additionally, predicted patterns for LNC and wood density did not match other maps (Figure 2; Butler et al., 2017; Moreno‐Martínez et al., 2018; Van Bodegom et al., 2014), but, given the poor predictive accuracy of the LNC models, we consider our LNC predictions unreliable (Figure 1).

Whenever trait predictions among different studies disagree, it is difficult to conclude which predictions are more reliable. One option is to validate global trait predictions against regional trait maps. For example, we found wood density to be higher in the east of the Amazon region compared with the north‐west and south‐west (Figure 2g), as was found by Baker et al. (2004). However, the paucity of regional trait maps makes this validation method impractical. Another option is to consider model predictive performances and the applicability domain to infer reliability of predictions. Unfortunately, the lack of quantification and indication of these in previous studies prevents us from drawing any conclusions about which predictions are more accurate.

### 4.2 Ecological evaluation of global trait predictions

In general, we found community mean SLA, plant height and wood density to vary more with climatic factors than with soil characteristics (Figure 3). This might reflect the greater variation in soil characteristics at smaller scales compared with climatic variables (Supporting Information Figure S11). Furthermore, all predicted trait combinations and correlations were realistic.

Our results confirmed most of the expected relationships between SLA and the environmental variables (Table 1; Figure 4). However, we found SLA to decrease with soil CEC and to show a flat response to soil pH, whereas we expected SLA to increase with soil fertility because less durable structures are thought to be maintained with higher soil fertility (Table 1; Figure 4). The flat response of SLA with soil pH might be explained by a high nutrient turnover rate in areas of low soil fertility (Vitousek & Sanford, 1986), although it might also be simply that SLA does not respond to changes in soil pH (Firn et al., 2019).

The expected responses of LNC to the environmental variables were not found (Table 1; Figure 4). The partial responses of LNC showed great variation over small environmental ranges, possibly owing to the tendency of machine learning models to over‐fit. Together with the low predictive performance of the models, this might reflect that LNC responds primarily to small‐scale environmental variation, whereas our models make predictions at a coarser resolution (55 km × 55 km). Our results thus support the deviating responses of LNC to environmental variables at the species level (Maire et al., 2015; Ordoñez et al., 2009; Reich & Oleksyn, 2004; Reich et al., 1996). Additionally, LNC might be highly variable in relationship to multiple environmental factors, leading to non‐universal adaptations to the predictors in our model (Bloomfield et al., 2018; Reich & Oleksyn, 2004). This variability makes it difficult to interpret LNC responses to large‐scale environmental gradients biologically, especially given that they will vary depending on whether intraspecific variation is considered or not (Albert et al., 2010). We conclude that it is not possible to predict LNC distributions reliably at this extent and resolution.

Our results confirmed the expected relationships between average community height and Tmin and PrecDryQ (Table 1; Figure 4). However, we found mean plant height to decrease with HumInd (Table 1; Figure 4). This might indicate that shorter vegetation (e.g., grasses, herbs and shrubs) is more abundant in more humid environments (i.e., higher annual rainfall and/or lower potential evapotranspiration). The unexpected increase in height with PrecSeas (Table 1; Figure 4) can be explained by the fact that many species in areas with seasonal rainfall can shed leaves, which makes the deciduous vegetation less vulnerable to cavitation compared with evergreen vegetation, allowing it to grow taller than expected. Furthermore, taller plants are better able to reach soil water reserves owing to deeper root systems (Borchert, 1998; Canadell et al., 1996).

Our results confirmed the expected relationships between wood density and Tmin, HumInd and soil CEC (Table 1; Figure 4). Both the increase in wood density with increasing Tmin and the unexpected flat response with PrecSeas might reflect that colder areas with less seasonal precipitation are dominated by soft‐wooded gymnosperms, whereas warmer areas with higher seasonal precipitation are dominated by angiosperms with generally denser wood (Swenson & Enquist, 2007), although xylem vulnerability for frost‐induced cavitation decreases with wood density (Reich, 2014). We expected a decrease in wood density with soil CEC and soil pH because higher soil fertility is expected to sustain higher growth rates. Although the overall trend confirmed our expectation, the response to pH was very limited, and at the lower end of the CEC gradient, wood density showed a small increase. This might indicate that extreme limitation to accessible soil nutrients cannot sustain high wood density, whereas low fertility but not nutrient‐limited environments promote slow growth, resulting in high wood density (Table 1). Given that no global trait–environment relationship has been described for wood density (Moles, 2018), these patterns should be investigated further.

Literature reports that some relationships between traits and environmental variables may vary with leaf habit, plant growth forms and photosynthetic pathway at the local scale (e.g., Šímová et al., 2018). Additionally, trait–environment relationships are affected by factors other than climate and soil properties, such as land‐use type and disturbance (e.g., Chen et al., 2018). However, we expect that these factors are of limited relevance at the biological scale (communities instead of species), spatial resolution and extent considered, where environmental filtering effects are expected to be much less confounded by biotic interactions and fine‐grain disturbances (Pearson & Dawson, 2003).

### 4.3 Evaluation of uncertainty

#### 4.3.1 Data quality

We found that including intraspecific trait variation contributed to improve the predictability of traits (Figure 5; Supporting Information Table S7). This improvement in trait predictions at the global scale highlights the importance of considering intraspecific trait variation over and above the conclusion from small‐scale studies that intraspecific trait variation contributes greatly to community trait variation (Albert et al., 2010; Bloomfield et al., 2018; Poorter, Castilho, Schietti, Oliveira, & Costa, 2018; Siefert et al., 2015). This indicates that widespread species are likely to show adaptability in their traits, changing them in order to optimize performance for different environments. Additionally, our results emphasize the need to build community trait models on a representative sample of species (Figure 5; Supporting Information Table S7). Moreover, high‐quality data not only improve models statistically, but also theoretically lead to different results (Poorter et al., 2018). Thus, the inclusion of intraspecific trait variation of species representative of the local vegetation should be preferred when the aim is to predict plant community mean trait values.

#### 4.3.2 Applicability domain

The strict selection criteria we set greatly reduced the amount of available community trait data (Supporting Information Table S2). Nevertheless, our dataset covered the major part of the global terrestrial environmental space, indicating a wide applicability domain of our models (Figure 6). However, trait predictions should be interpreted carefully for deserts, the Arctic and tropical islands because of the high variation in predictions between models, and for mountainous areas because of the high environmental variation within a grid cell. Furthermore, predictions for wood density are extrapolated to a larger extent in comparison to other traits. A reason for the fewer community mean data points for wood density is that species mean values are generally considered appropriate because interspecific variation in wood density is larger than the intraspecific variation; therefore, new wood density data are rarely collected (Siefert et al., 2015). Additionally, most wood density measures available were collected before 1980, meaning that taking global species averages of wood density does not consider any evolutionary responses to changes in the local environment. Finally, wood density is rarely reported because of the challenges of measuring it in small shrubs and low vegetation (Perez‐Harguindeguy et al., 2013).

#### 4.3.3 Model selection

The selection of a specific modelling technique greatly affected the ability to predict traits, and no single “best” model could be identified (Figure 1). Nevertheless, the ensemble forecasting approach as used here is equal to or better at predicting each trait than any individual model. Additionally, this ensemble approach can be used to retrieve a single prediction relying mostly on the best‐performing models, while considering the variation in predictions between models with similar support. Furthermore, large variation in values predicted by different models, such as for plant height in the Sahara and the North of India, can be used as an indication of uncertainty. Thus, ensemble forecasting is not only better in predicting traits compared with single models, it also reduces the uncertainty attributable to subjective model technique selection and it enables the quantification and mapping of the uncertainty attributable to divergent model predictions. Finally, it allows for one general modelling approach for multiple traits.

### 4.4 Reliability of global plant trait predictions

Our results suggest that plant community traits can be predicted reliably at the global scale when using an ensemble approach with high‐quality data (i.e., including intraspecific trait variation and a representative species sample). We show that intraspecific trait variation and the representativeness of species considered in a community are important factors to consider, even at the global scale, and that an ensemble forecasting approach helps to deal with and quantify multiple types of uncertainty. Based on these results, we recommend the systematic and careful selection of data and modelling techniques for trait–environment models, and more routine assessment of their reliability based on model predictive performance, applicability domain, model uncertainty and realism of predicted trait combinations. Such systematic presentation of validation results and applicability domain in studies presenting predictions of spatial patterns of community mean traits will enhance our ability to build upon previous modelling attempts and improve our understanding of trait–environment relationships.

Our approach also led to new insights, such as the unexpected increase of community height with the seasonality of precipitation, but the lack of proper model assessment by previous studies limits our ability to draw any objective conclusions on the observed differences in trait responses. We suggest that higher predictive accuracy can be achieved for traits that respond primarily to large‐scale environmental factors, such as specific leaf area, whereas predictive accuracy at this extent and resolution would be lower for traits such as LNC that might respond primarily to small‐scale environmental variation. Nevertheless, the results of our trait models can improve the mechanistic understanding of global plant trait–environment relationships and contribute to answering the central question in functional ecology of the predictability of traits by the environment. Our assessment opens a new avenue to test impacts of global change on trait distributions and, eventually, on plant communities. Ultimately, the results of our global trait models are contingent on the quality and distributions of the trait data. Modelling efforts will be enriched greatly by improving data collection or data availability in areas where no or few data are available and for locations characterized by unique environmental conditions.

## BIOSKETCH

**Coline Boonman** is a PhD candidate at the Radboud University in The Netherlands, and her research focuses on local‐scale variation and macroecological patterns of plant functional traits. Her main interests are the adaptability of plants to different environments and the effect of natural and anthropogenic disturbances on vegetation patterns.

## Supporting information

Appendix S1‐S11

## Data Availability

Community mean trait data used in this paper and the predicted trait distributions maps generated in this study are provided via FigShare, as are all R scripts regarding data analyses (http://doi.org/10.6084/m9.figshare.11559852). Owing to regulations of the major trait database used, the raw data cannot be shared openly.

## APPENDIX 1 DATA SOURCES

Ackerly, D. (2004). Functional strategies of chaparral shrubs in relation to seasonal water deficit and disturbance. *Ecological Monographs*, *74*, 25–44.

Adler, P. B., Milchunas, D. G., Lauenroth, W. K., Sala, O. E., & Burke, I. C. (2004). Functional traits of graminoids in semi‐arid steppes: A test of grazing histories. *Journal of Applied Ecology*, *41*, 653–663.

Baddeley, J. A., Woodin, S. J., & Alexander, I. J. (1994). Effects of increased nitrogen and phosphorus availability on the photosynthesis and nutrient relations of three Arctic dwarf Shrubs from Svalbard. *Functional Ecology*, *8*, 676–685.

Baraloto, C., Paine, C. E. T., Poorter, L., Beauchene, J., Bonal, D., Domenach, A. M., … Chave, J. (2010). Decoupled leaf and stem economics in rain forest trees. *Ecology Letters*, *13*, 1338–1347.

Barrett, R. T. S., Hollister, R. D., Oberbauer, S. F., & Tweedie, C. E. (2015). Arctic plant responses to changing abiotic factors in northern Alaska. *American Journal of Botany*, 102, 2020–2031.

Baruch, Z., & Goldstein, G. (1999). Leaf construction cost, nutrient concentration, and net CO_2_ assimilation of native and invasive species in Hawaii. *Oecologia*, *121*, 183–192.

Bassow, S. L., & Bazzaz, F. A. (1997). Intra‐ and inter‐specific variation in canopy photosynthesis in a mixed deciduous forest. *Oecologia*, *109*, 507–515.

Berner, L. T., Alexander, H. D., Loranty, M. M., Ganzlin, P., Mack, M. C., Davydov, S. P., & Goetz, S. J. (2015). Biomass allometry for alder, dwarf birch, and willow in boreal forest and tundra ecosystems of far northeastern Siberia and north‐central Alaska. *Forest Ecology and Management*, *337*, 110–118.

Bjorkman, A. D., Elmendorf, S. C., Beamish, A. L., Vellend, M., & Henry, G. H. R. (2015). Contrasting effects of warming and increased snowfall on Arctic tundra plant phenology over the past two decades. *Global Change Biology*, *21*, 4651–4661.

Blonder, B., Buzzard, V., Simova, I., Sloat, L., Boyle, B., Lipson, R., … Enquist, B. J. (2012). The leaf‐area shrinkage effect can bias paleoclimate and ecology research. *American Journal of Botany*, *99*, 1756–1763.

Blonder, B., Vasseur, F., Violle, C., Shipley, B., Enquist, B. J., & Vile, D. (2015). Testing models for the leaf economics spectrum with leaf and whole‐plant traits in *Arabidopsis thaliana*. *AoB PLANTS*, *7*, plv049.

Blonder, B., Violle, C., Bentley, L. P., & Enquist, B. J. (2011). Venation networks and the origin of the leaf economics spectrum. *Ecology Letters*, *14*, 91–100.

Blonder, B., Violle, C., & Enquist, B. J. (2013). Assessing the causes and scales of the leaf economics spectrum using venation networks in *Populus tremuloides*. *Journal of Ecology*, *101*, 981–989.

Bloomfield, K. J., Cernusak, L. A., Eamus, D., Ellsworth, D. S., Prentice, I. C., Wright, I. J., … Atkin, O. K. (2018). A continental‐scale assessment of variability in leaf traits: Within species, across sites and between seasons. *Functional Ecology*, *32*, 1492–1506.

Bond‐Lamberty, B., Wang, C., & Gower, S. T. (2002). Aboveground and belowground biomass and sapwood area allometric equations for six boreal tree species of northern Manitoba. *Canadian Journal of Forest Research*, *32*, 1441–1450.

Bond‐Lamberty, B., Wang, C., Gower, S. T., & Norman, J. (2002). Leaf area dynamics of a boreal black spruce fire chronosequence. *Tree Physiology*,* 22*, 993–1001.

Bongers, F., & Pompa, J. (1990). Leaf characteristics of the tropical rain forest flora of Los Tuxtlas, Mexico. *Botanical Gazette*, *151*, 354–365.

Brown, K. A., Johnson, S. E., Parks, K. E., Holmes, S. M., Ivoandry, T., Abram, N. K., … Wright, P. C. (2013). Use of provisioning ecosystem services drives loss of functional traits across land use intensification gradients in tropical forests in Madagascar. *Biological Conservation*, *161*, 118–127.

Butterfield, B. J., & Briggs, J. M. (2011). Regeneration niche differentiates functional strategies of desert woody plant species. *Oecologia*, *165*, 477–487.

Campetella, G., Botta‐Dukát, Z., Wellstein, C., Canullo, R., Gatto, S., Chelli, S., … Bartha, S. (2011). Patterns of plant trait–environment relationships along a forest succession chronosequence. *Agriculture, Ecosystems and Environment*,* 145*, 38–48.

Carbognani, M., Petraglia, A., & Tomaselli, M. (2014). Warming effects and plant trait control on the early‐decomposition in alpine snowbeds. *Plant and Soil*, *376*, 277–290.

Cerabolini, B. E. L., Brusa, G., Ceriani, R. M., de Andreis, R., Luzzaro, A., & Pierce, S. (2010). Can CSR classification be generally applied outside Britain? *Plant Ecology*, *210*, 253–261.

Chapin, F. S. I., & Shaver, G. R. (1985). Individualistic growth response of tundra plant species to environmental manipulations in the field. *Ecology*, *66*, 564–576.

Chapin, F. S. I., Shaver, G. R., Giblin, A. E., Nadelhoffer, K. J., & Laundre, J. A. (1995). Responses of Arctic tundra to experimental and observed changes in climate. *Ecology*, *76*, 694–711.

Chen, Y., Han, W., Tang, L., Tang, Z., & Fang, J. (2013). Leaf nitrogen and phosphorus concentrations of woody plants differ in responses to climate, soil and plant growth form. *Ecography*, *36*, 178–184.

Cornelissen, J. H. C., Cerabolini, B., Castro‐Díez, P., Villar‐Salvador, P., Montserrat‐Martí, G., Puyravaud, J. P., … Aerts, R. (2003). Functional traits of woody plants: Correspondence of species rankings between field adults and laboratory‐grown seedlings? *Journal of Vegetation Science*, *14*, 311–322.

Cornelissen, J. H. C., Diez, P. C., & Hunt, R. (1996). Seedling growth, allocation and leaf attributes in a wide range of woody plant species and types. *Journal of Ecology*, *84*, 755–765.

Cornelissen, J. H. C., Quested, H. M., Gwynn‐Jones, D., Van Logtestijn, R. S. P., De Beus, M. A. H., Kondratchuk, A., … Aerts, R. (2004). Leaf digestibility and litter decomposability are related in a wide range of subarctic plant species and types. *Functional Ecology*, *18*, 779–786.

Cornwell, W. K., & Ackerly, D. D. (2009). Community assembly and shifts in plant trait distributions across an environmental gradient in coastal California. *Ecological Monographs*, *79*, 109–126.

Craine, J. M., & Towne, E. G. (2010). High leaf tissue density grassland species consistently more abundant across topographic and disturbance contrasts in a North American tallgrass prairie. *Plant and Soil*, *337*, 193–203.

Craine, J. M., Towne, E. G., Ocheltree, T. W., & Nippert, J. B. (2012). Community traitscape of foliar nitrogen isotopes reveals N availability patterns in a tallgrass prairie. *Plant and Soil*, *356*, 395–403.

Craven, D., Braden, D., Ashton, M. S., Berlyn, G. P., Wishnie, M., & Dent, D. (2007). Between and within‐site comparisons of structural and physiological characteristics and foliar nutrient content of 14 tree species at a wet, fertile site and a dry, infertile site in Panama. *Forest Ecology and Management*, *238*, 335–346.

de Araujo, A. C., Ometto, J. P. H. B., Dolman, A. J., Kruijt, B., Waterloo, M. J., & Ehleringer, J. R. (2012). LBA‐ECO CD‐02 C and N isotopes in leaves and atmospheric CO2, Amazonas, Brazil, Data set., Oak Ridge National Laboratory Distributed Active A. Retrieved from http://doi.org/10.3334/ORNLDAAC/1097

Devictor, V., Mouillot, D., Meynard, C., Jiguet, F., Thuiller, W., & Mouquet, N. (2010). Spatial mismatch and congruence between taxonomic, phylogenetic and functional diversity: The need for integrative conservation strategies in a changing world. *Ecology Letters*, *13*, 1030–1040.

Diemer, M. (1998). Leaf lifespans of high‐elevation, aseasonal Andean shrub species in relation to leaf traits and leaf habit. *Global Ecology and Biogeography Letters*, *7*, 457–465.

Diemer, M. (1998). Life span and dynamics of leaves of herbaceous perennials in high‐elevation environments: “News from the elephant's leg”. *Functional Ecology*, *12*, 413–425.

Diemer, M., Körner, C., & Prock, S. (1992). Leaf life spans in wild perennial herbaceous plants: A survey and attempts at a functional interpretation. *Oecologia*, *89*, 10–16.

Ellsworth, D. S., & Reich, P. B. (1993). Canopy structure and vertical patterns of photosynthesis and related leaf traits in a deciduous forest. *Oecologia*, *96*, 169–178.

Eskelinen, A., Kaarlejärvi, E., & Olofsson, J. (2017). Herbivory and nutrient limitation protect warming tundra from lowland species' invasion and diversity loss. *Global Change Biology*, *23*, 245–255.

Figueira, A. M. E. S., Miller, S. D., de Sousa, C. A. D., Menton, M. C., Maia, A. R., da Rocha, H. R., & Goulden, M. L. (2008). Effects of selective logging on tropical forest tree growth. *Journal of Geophysical Research*, *113*, G00B05.

Frenette‐Dussault, C., Shipley, B., Léger, J. F., Meziane, D., & Hingrat, Y. (2012). Functional structure of an arid steppe plant community reveals similarities with Grime's C‐S‐R theory. *Journal of Vegetation Science*, *23*, 208–222.

Freschet, G. T., Cornelissen, J. H. C., van Logtestijn, R. S. P., & Aerts, R. (2010). Evidence of the “plant economics spectrum” in a subarctic flora. *Journal of Ecology*, *98*, 362–373.

Fyllas, N. M., Patino, S., Baker, T. R., Bielefeld Nardoto, G., Martinelli, L. A., Quesada, C. A., … Lloyd, J. (2009). Basin‐wide variations in foliar properties of Amazonian forest: Phylogeny, soils and climate. *Biogeosciences*, *6*, 2677–2708.

Garnier, E., Laurent, G., Bellmann, A., Debain, S., Berthelier, P., Ducout, B., … Navas, M.‐L. (2001). Consistency of species ranking based on functional leaf traits. *New Phytologist*, *152*, 69–83.

Garnier, E., Lavorel, S., Ansquer, P., Castro, H., Cruz, P., Dolezal, J., … Zarovali, M. P. (2007). Assessing the effects of land‐use change on plant traits, communities and ecosystem functioning in grasslands: A standardized methodology and lessons from an application to 11 European sites. *Annals of Botany 99*, 967–985.

Gleason, S. M., Butler, D. W., Ziemińska, K., Waryszak, P., & Westoby, M. (2012). Stem xylem conductivity is key to plant water balance across Australian angiosperm species. *Functional Ecology*, *26*, 343–352.

Good, M. K., Morgan, J. W., Venn, S., & Green, P. (2019). Timing of snowmelt affects species composition via plant strategy filtering. *Basic and Applied Ecology*, *35*, 54–62.

Grau, O., Ninot, J. M., Pérez‐Haase, A., & Callaghan, T. V. (2014). Plant co‐existence patterns and high‐arctic vegetation composition in three common plant communities in north‐east Greenland. *Polar Research*, *33*, 19235.

Gutiérrez, A. G., Armesto, J. J., & Aravena, J. C. (2004). Disturbance and regeneration dynamics of an old‐growth North Patagonian rain forest in Chiloé Island, Chile. *Journal of Ecology*, *92*, 598–608.

Gutiérrez, A. G., & Huth, A. (2012). Successional stages of primary temperate rainforests of Chiloé Island, Chile. *Perspectives in Plant Ecology, Evolution and Systematics*, *14*, 243–256.

Guy, A. L., Mischkolz, J. M., & Lamb, E. G. (2013). Limited effects of simulated acidic deposition on seedling survivorship and root morphology of endemic plant taxa of the Athabasca Sand Dunes in well‐watered greenhouse trials. *Botany*, *91*, 176–181.

Han, Q., Kawasaki, T., Nakano, T., & Chiba, Y. (2004). Spatial and seasonal variability of temperature responses of biochemical photosynthesis parameters and leaf nitrogen content within a *Pinus densiflora* crown. *Tree Physiology*, *24*, 737–744.

Han, W., Chen, Y., Zhao, F. J., Tang, L., Jiang, R., & Zhang, F. (2012). Floral, climatic and soil pH controls on leaf ash content in China's terrestrial plants. *Global Ecology and Biogeography*, *21*, 376–382.

Harper, K. A., Lavallee, A. A., & Dodonov, P. (2018). Patterns of shrub abundance and relationships with other plant types within the forest–tundra ecotone in northern Canada. *Arctic Science*, *4*, 691–709.

Hikosaka, K., & Hirose, T. (2000). Photosynthetic nitrogen‐use efficiency in evergreen broad‐leaved woody species coexisting in a warm‐temperate forest. *Tree Physiology*, *20*, 1249–1254.

Hikosaka, K., Nagamatsu, D., Ishii, H. S., & Hirose, T. (2002). Photosynthesis–nitrogen relationships in species at different altitudes on Mount Kinabalu, Malaysia. *Ecological Research*, *17*, 305–313.

Hudson, J. M. G., Henry, G. H. R., & Cornwell, W. K. (2011). Taller and larger: Shifts in Arctic tundra leaf traits after 16 years of experimental warming. *Global Change Biology*, *17*, 1013–1021.

Iturrate‐Garcia, M., O'Brien, M. J., Khitun, O., Abiven, S., Niklaus, P. A., & Schaepman‐Strub, G. (2016). Interactive effects between plant functional types and soil factors on tundra species diversity and community composition. *Ecology and Evolution*, *6*, 8126–8137.

Jurik, T. W. (1986). Temporal and spatial patterns of specific leaf weight in successional northern hardwood tree species. *American Journal of Botany*, *73*, 1083–1092.

Jurik, T. W., Weber, J. A., & Gates, D. M. (1988). Effects of temperature and light on photosynthesis of dominant species of a northern hardwood forest. *Botanical Gazette*, *149*, 203–208.

Kaarlejärvi, E., Baxter, R., Hofgaard, A., Hytteborn, H., Khitun, O., Molau, U., … Olofsson, J. (2012). Effects of sarming on shrub abundance and chemistry drive ecosystem‐level changes in a forest–tundra ecotone. *Ecosystems, 15*, 1219–1233.

Kaarlejärvi, E., Eskelinen, A., & Olofsson, J. (2017). Herbivores rescue diversity in warming tundra by modulating trait‐dependent species losses and gains. *Nature Communications*, *8*, 419.

Kattge, J., Knorr, W., Raddatz, T., & Wirth, C. (2009). Quantifying photosynthetic capacity and its relationship to leaf nitrogen content for global‐scale terrestrial biosphere models. *Global Change Biology*, *15*, 976–991.

Kazakou, E., Vile, D., Shipley, B., Gallets, C., & Garnier, E. (2006). Co‐variations in litter decomposition, leaf traits and plant growth in species from a Mediterranean old‐field succession. *Functional Ecology*, *20*, 21–30.

Kichenin, E., Wardle, D. A., Peltzer, D. A., Morse, C. W., & Freschet, G. T. (2013). Contrasting effects of plant inter‐ and intraspecific variation on community‐level trait measures along an environmental gradient. *Functional Ecology*, *27*, 1254–1261.

Klady, R. A., Henry, G. H. R., & Lemay, V. (2011). Changes in high arctic tundra plant reproduction in response to long‐term experimental warming. *Global Change Biology*, *17*, 1611–1624.

Kraft, N. J. B., Valencia, R., & Ackerly, D. D. (2008). Functional traits and niche‐based tree community assembly in an Amazonian forest. *Science*, *322*, 580–582.

Kudo, G. (1996). Intraspecific variation of leaf traits in several deciduous species in relation to length of growing season. *Écoscience*, *3*, 483–489.

Kudo, G., Molau, U., & Wada, N. (2001). Leaf‐trait variation of tundra plants along a climatic gradient: An integration of responses in evergreen and deciduous species. *Arctic, Antarctic, and Alpine Research*, *33*, 181–190.

Kurokawa, H., & Nakashizuka, T. (2008). Leaf herbivory and decomposability in a Malaysian tropical rain forest. *Ecology*, *89*, 2645–2656.

Laughlin, D. C., Fulé, P. Z., Huffman, D. W., Crouse, J., & Laliberté, E. (2011). Climatic constraints on trait‐based forest assembly. *Journal of Ecology*, *99*, 1489–1499.

Laughlin, D. C., Leppert, J. J., Moore, M. M., & Sieg, C. H. (2010). A multi‐trait test of the leaf‐height‐seed plant strategy scheme with 133 species from a pine forest flora. *Functional Ecology*, *24*, 493–501.

Little, C. J., Cutting, H., Alatalo, J., & Cooper, E. J. (2017). Short‐term herbivory has long‐term consequences in warmed and ambient high Arctic tundra. *Environmental Research Letters*, *12*, 025001.

McAllister, C. A., Knapp, A. K., & Maragni, L. A. (1998). Is leaf‐level photosynthesis related to plant success in a highly productive grassland? *Oecologia*, *117*, 40–46.

Medlyn, B., Badeck, F.‐W., De Pury, D., Barton, C., Broadmeadow, M., Ceulemans, R., … Jarvis, P. (1999). Effects of elevated [CO_2_] on photosynthesis in European forest species: A meta‐analysis of model parameters. *Plant Cell and Environment*, *22*, 1475–1495.

Meir, P., Levy, P. E., Grace, J., & Jarvis, P. G. (2007). Photosynthetic parameters from two contrasting woody vegetation types in West Africa. *Plant Ecology*, *192*, 277–287.

Messier, J., McGill, B. J., & Lechowicz, M. J. (2010). How do traits vary across ecological scales? A case for trait‐based ecology. *Ecology Letters*, *13*, 838–848.

Midgley, J. J., van Wyk, G. R., & Everard, D. A. (1995). Leaf attributes of South African forest species. *African Journal of Ecology*, *33*, 160–168.

Milla, R., & Reich, P. B. (2011). Multi‐trait interactions, not phylogeny, fine‐tune leaf size reduction with increasing altitude. *Annals of Botany*, *107*, 455–465.

Missio, F., Higuchi, P., Silva, A., Longhi, S., Salami, B., Dalla Rosa, A., … Bento, M. (2016). Trade‐offs and spatial variation of functional traits of tree species in a subtropical forest in southern Brazil. *IForest, 9*, 855–859.

Mitchell, K. A., Bolstad, P. V, & Vose, J. M. (1999). Interspecific and environmentally induced variation in foliar dark respiration among eighteen southeastern deciduous tree species. *Tree Physiology*, *19*, 861–870.

Mooney, H. A., Field, C., Gulmon, S. L., & Bazzaz, F. A. (1981). Photosynthetic capacity in relation to leaf position in desert versus old‐field annuals. *Oecologia*, *50*, 109–112.

Morales, D., Gonzalez‐Rodriguez, A. M., & Cermak, J. (1996). Laurel forests in Tenerife, Canary Islands: The vertical profiles of leaf characteristics. *Phyton*, *36*, 251–263.

Mörsdorf, M. A., Baggesen, N. S., Yoccoz, N. G., Michelsen, A., Elberling, B., Ambus, P. L., & Cooper, E. J. (2019). Deepened winter snow significantly influences the availability and forms of nitrogen taken up by plants in High Arctic tundra. *Soil Biology and Biochemistry*, *135*, 222–234.

Müller, S. C., Overbeck, G. E., Pfadenhauer, J., & Pillar, V. D. (2007). Plant functional types of woody species related to fire disturbance in forest–grassland ecotones. *Plant Ecology*, *189*, 1–14.

Nauta, A. L., Heijmans, M. M. P. D., Blok, D., Limpens, J., Elberling, B., Gallagher, A., … Berendse, F. (2015). Permafrost collapse after shrub removal shifts tundra ecosystem to a methane source. *Nature Climate Change*, *5*, 67–70.

Niinemets, Ü. (1999). Components of leaf dry mass per area – thickness and density – alter leaf photosynthetic capacity in reverse directions in woody plants. *New Phytologist*, *144*, 35–47.

Ogaya, R., & Peñuelas, J. (2003). Comparative field study of *Quercus ilex* and *Phillyrea latifolia*: Photosynthetic response to experimental drought conditions. *Environmental and Experimental Botany*, *50*, 137–148.

Ordoñez, J. C., van Bodegom, P. M., Witte, J.‐P. M., Bartholomeus, R. P., van Hal, J. R., & Aerts, R. (2010). Plant strategies in relation to resource supply in mesic to wet environments: Does theory mirror nature? *The American Naturalist*, *175*, 225–239.

Overbeck, G. E., Müller, S. C., Pillar, V. D., & Pfadenhauer, J. (2005). Fine‐scale post‐fire dynamics in southern Brazilian subtropical grassland. *Journal of Vegetation Science*, *16*, 655–664.

Parada, T. J., Jara, C. V., & Lusk, C. H. (2003). Distribución de alturas máximas de especies en rodales antiguos de selva Valdiviana, Parque Nacional Puyehue. *Bosque*, *24*, 63–67.

Peco, B., De Pablos, I., Traba, J., & Levassor, C. (2005). The effect of grazing abandonment on species composition and functional traits: The case of dehesa grasslands. *Basic and Applied Ecology*, *6*, 175–183.

Penuelas, J., Sardans, J., Llusià, J., Owen, S. M., Carnicer, J., Giambelluca, T. W., … Niinemets, Ü. (2010). Faster returns on ‘leaf economics’ and different biogeochemical niche in invasive compared with native plant species. *Global Change Biology*, *16*, 2171–2185.

Pickering, C. M., & Venn, S. (2013). Increasing the resilience of Australian alpine flora to climate change and assosiated threats: A plant functional traits approach. Australia: National Climate Change Adaptation Research Facility.

Poorter, L. (2009). Leaf traits show different relationships with shade tolerance in moist versus dry tropical forests. *New Phytologist*, *181*, 890–900.

Poorter, L., & Bongers, F. (2011). Leaf traits are good predictors of plant performance across 53 rain forest species. *Ecology*, *87*, 1733–1743.

Powers, J. S., & Tiffin, P. (2010). Plant functional type classifications in tropical dry forests in Costa Rica: Leaf habit versus taxonomic approaches. *Functional Ecology*, *24*, 927–936.

Prentice, I. C., Meng, T., Wang, H., Harrison, S. P., Ni, J., & Wang, G. (2011). Evidence of a universal scaling relationship for leaf CO_2_. *New Phytologist*, *190*, 169–180.

Price, C. A., & Enquist, B. J. (2007). Scaling mass and morphology in leaves: An extension of the WBE model. *Ecology*, *88*, 1132–1141.

Pyankov, V. I., Kondratchuk, A. V., & Shipley, B. (1999). Leaf structure and specific leaf mass: the alpine desert plants of the Eastern Pamirs, Tadjikistan. *New Phytologist*, *143*, 131–142.

Quested, H. M., Cornelissen, J. H. C., Press, M. C., Callaghan, T. V, Aerts, R., Trosien, F., … Jonasson, S. E. (2003). Decomposition of sub‐Arctic plants with differing nitrogen economies: A functional role for hemiparasites. *Ecology*,* 84*, 3209–3221.

Read, Q. D., Henning, J. A., Classen, A. T., & Sanders, N. J. (2018). Aboveground resilience to species loss but belowground resistance to nitrogen addition in a montane plant community. *Journal of Plant Ecology*, *11*, 351–363.

Reich, P. B., Ellsworth, D. S., Walters, M. B., Vose, J. M., Greshyam, C., Volin, J. C., … Bowman, W. D. (1999). Generelity of leaf trait relationships: A test across six biomes. *Ecology*, *80*, 1955–1969.

Reich, P. B., Oleksyn, J., & Wright, I. J. (2009). Leaf phosphorus influences the photosynthesis–nitrogen relation: A cross‐biome analysis of 314 species. *Oecologia*, *160*, 207–212.

Rumpf, S. B., Semenchuk, P. R., Dullinger, S., & Cooper, E. J. (2014). Idiosyncratic responses of high arctic plants to changing snow regimes. *PLoS ONE*, *9*, e86281.

Sandel, B., Corbin, J. D., & Krupa, M. (2011). Using plant functional traits to guide restoration: A case study in California coastal grassland. *Ecosphere*, *2*, 1–16.

Schlesinger, W. H., DeLucia, E. H., & Billings, W. D. (1989). Nutrient‐use efficiency of woody plants on contrasting soils in the western Great Basin, Nevada. *Ecology*, *70*, 105–113.

Simpson, A. H., Richardson, S. J., & Laughlin, D. C. (2016). Soil–climate interactions explain variation in foliar, stem, root and reproductive traits across temperate forests. *Global Ecology and Biogeography*, *25*, 964–978.

Sobrado, M. A. (1991). Cost–benefit relationships in deciduous and evergreen leaves of tropical dry forest species. *Functional Ecology*, *5*, 608–616.

Soudzilovskaia, N. A., Elumeeva, T. G., Onipchenko, V. G., Shidakov, I. I., Salpagarova, F. S., Khubiev, A. B., … Cornelissen, J. H. C. (2013). Functional traits predict relationship between plant abundance dynamic and long‐term climate warming. *Proceedings of the National Academy of Sciences USA*, *110*, 18180–18184.

Spasojevic, M. J., & Suding, K. N. (2012). Inferring community assembly mechanisms from functional diversity patterns: The importance of multiple assembly processes. *Journal of Ecology*, *100*, 652–661.

Speed, J. D. M., Austrheim, G., Hester, A. J., & Mysterud, A. (2010). Experimental evidence for herbivore limitation of the treeline. *Ecology*, *91*, 3414–3420.

Street, L. E., Subke, J.‐A., Baxter, R., Dinsmore, K. J., Knoblauch, C., & Wookey, P. A. (2018). Ecosystem carbon dynamics differ between tundra shrub types in the western Canadian Arctic. *Environmental Research Letters*, *13*, 084014.

van der Sande, M. T., Arets, E. J. M. M., Peña‐Claros, M., De Avila, A. L., Roopsind, A., Mazzei, L., … Poorter, L. (2016). Old‐growth Neotropical forests are shifting in species and trait composition. *Ecological Monographs*, *86*, 228–243.

van der Sande, M. T., Arets, E. J. M. M., Peña‐Claros, M., Hoosbeek, M. R., Cáceres‐Siani, Y., van der Hout, P., & Poorter, L. (2018). Soil fertility and species traits, but not diversity, drive productivity and biomass stocks in a Guyanese tropical rainforest. *Functional Ecology*, *32*, 461–474.

van der Sande, M. T., Peña‐Claros, M., Ascarrunz, N., Arets, E. J. M. M., Licona, J. C., Toledo, M., & Poorter, L. (2017). Abiotic and biotic drivers of biomass change in a Neotropical forest. *Journal of Ecology*, *105*, 1223–1234.

Villar, R., & Merino, J. (2001). Comparison of leaf construction costs in woody species with differing leaf life‐spans in contrasting ecosystems. *New Phytologist*, *151*, 213–226.

Von Holle, B., & Simberloff, D. (2004). Testing Fox's assembly rule: does plant invasion depend on recipient community structure? *Oikos*, *105*, 551–563.

Vowles, T., Gunnarsson, B., Molau, U., Hickler, T., Klemedtsson, L., & Björk, R. G. (2017). Expansion of deciduous tall shrubs but not evergreen dwarf shrubs inhibited by reindeer in Scandes mountain range. *Journal of Ecology*, *105*, 1547–1561.

Whitehead, D., Walcroft, A. S., Scott, N. A., Townsend, J. A., Trotter, C. M., & Rogers, G. N. D. (2004). Characteristics of photosynthesis and stomatal conductance in the shrubland species manuka (*Leptospermum scoparium*) and kanuka (*Kunzea ericoides*) for the estimation of annual canopy carbon uptake. *Tree Physiology*, *24*, 795–804.

Williams, M., Shimabokuro, Y. E., & Rastetter, E. B. (2012). *LBA‐ECO CD‐09 Soil and vegetation characteristics, Tapajos National Forest, Brazil, Data set*. Retrieved from http://daac.ornl.gov. 10.3334/ORNLDAAC/1104

Williams‐Linera, G. (2000). Leaf demography and leaf traits of temperate‐deciduous and tropical evergreen‐broadleaved trees in a Mexican montane cloud forest. *Plant Ecology*, *149*, 233–244.

Wright, I. J., Reich, P. B., & Westoby, M. (2001). Strategy shifts in leaf physiology, structure and nutrient content between species of high‐ and low‐rainfall and high‐ and low‐nutrient habitats. *Functional Ecology*, *15*, 423–434.

Wright, I. J., Reich, P. B., Westoby, M., Ackerly, D. D., Baruch, Z., Bongers, F., … Gulias, J. (2004). The worldwide leaf economics spectrum. *Nature*, *428*, 821–827.

Wright, I. J., Westoby, M., & Reich, P. B. (2002). Convergence towards higher leaf mass per area in dry and nutrient‐poor habitats has different consequences for leaf life span. *Journal of Ecology*, *90*, 534–543.

Wright, S. J., Kitajima, K., Kraft, N. J. B., Reich, P. B., Wright, I. J., Bunker, D. E., … Zanne, A. E. (2010). Functional traits and the growth–mortality trade‐off in tropical trees. *Ecology*, *91*, 3664–3674.
