# Supplementary material for: Assessing the reliability of predicted plant trait distributions at the global scale
Source: Glob Ecol Biogeogr. 2020 Mar 20;29(6):1034–51. doi: 10.1111/geb.13086 (PMC7319484; doi:10.1111/geb.13086)
Supplement: Supplementary file 1 — Appendix S1‐S11 [file GEB-29-1034-s001.docx]

Supporting Information

Assessing the reliability of predicted plant trait distributions at the global scale

Authors:

Coline C.F. Boonman, Ana Benítez-López, Aafke M. Schipper, Wilfried Thuiller, Madhur Anand, Bruno E.L. Cerabolini, Johannes H.C. Cornelissen, Andres Gonzalez-Melo, Wesley N. Hattingh, Pedro Higuchi, Daniel C Laughlin, Vladimir G. Onipchenko, Josep Peñuelas, Lourens Poorter, Nadejda A. Soudzilovskaia, Mark A.J. Huijbregts, Luca Santini

Content:

Appendix S1. Original references

Appendix S2. Data selection and cleaning

Appendix S3. Community means

Appendix S4. Data distribution

Appendix S5. Considered environmental predictors

Appendix S6. Model parameterization

Appendix S7. Assessing the effect of including intra-specific trait variation and species representativeness

Appendix S8. Coefficient of variation of trait predictions

Appendix S9. Unrealistic predicted trait combinations

Appendix S10. Environmental coverage of trait data

Appendix S11. Global environmental variation

References

**Appendix S1.** Original references

We received data from TRY (Table S1.1), the Tundra Trait Team (TTT) (Table S1.2), and various other datasets both published (Bloomfield et al., 2018; J. H. C. Cornelissen et al., 2003; Gleason, Butler, Ziemińska, Waryszak, & Westoby, 2012; Kraft, Valencia, & Ackerly, 2008; Meir, Levy, Grace, & Jarvis, 2007; Simpson, Richardson, & Laughlin, 2016; van der Sande et al., 2017, 2018; Van Der Sande et al., 2016) and unpublished (Schamp, B.; Santini, L.).

**Table S1.1** List of references for TRY data that are included in this study. The list includes the dataset identification numbers as used in TRY and the original references per dataset and studies in datasets of the data that was included in our database. In the case of unpublished data, the criteria were checked with the dataset custodians. The full references of the original data sources can be found in Appendix S3.

| DatasetID | References |
| --- | --- |
| 1 | (A. J. H. C. Cornelissen, Diez, & Hunt, 1996; J. H. C. Cornelissen et al., 2004; Quested et al., 2003) |
| 12 | (Medlyn et al., 1999) |
| 20 | GLOPNET – Global Plant Trait Network Database (Ian J Wright et al., 2004) – Only data originating from the following references were included: (Baruch & Goldstein, 1999; M. Diemer, Körner, & Prock, 1992; M Diemer, 1998; Matthias Diemer, 1998; E. Garnier et al., 2001; K. Hikosaka & Hirose, 2000; Kouki Hikosaka, Nagamatsu, Ishii, & Hirose, 2002; Jurik, 1986; Kudo, 1996; Kudo, Molau, & Wada, 2001; Mcallister, Knapp, & Maragni, 1998; Midgley, van Wyk, & Everard, 1995; Mitchell, Bolstad, & Vose, 1999; Mooney, Field, Gulmon, & Bazzaz, 1981; Pyankov, Kondratchuk, & Shipley, 1999; Reich et al., 1999; Schlesinger, DeLucia, & Billings, 1989; Sobrado, 1991; Villar & Merino, 2001; Williams-linera, 2000; I. A. N. J. Wright, Westoby, & Reich, 2002; I J Wright, Reich, & Westoby, 2001) |
| 33 | (Poorter, 2009; Poorter & Bongers, 2011) |
| 34 | (Fyllas et al., 2009) |
| 45 | (Eric Garnier et al., 2007) |
| 50 | Leaf and Whole Plant Traits Database – Only data originating from the following references were included: (Kazakou, Vile, Shipley, Gallets, & Garnier, 2006) |
| 52 | (Kurokawa & Nakashizuka, 2008) |
| 55 | (Cornwell & Ackerly, 2009) |
| 65 | Overton, J. and Wright, I.J., unpublished data |
| 67 | Leaf Physiology Database (Jurik, Weber, & Gates, 1988; Kattge, Knorr, Raddatz, & Wirth, 2009) – Only data originating from the following references were included: (Bassow & Bazzaz, 1997; Ellsworth & Reich, 1993; Q. Han, Kawasaki, Nakano, & Chiba, 2004; Kouki Hikosaka et al., 2002; Whitehead et al., 2004) |
| 73 | (Chapin & Shaver, 1985; Chapin, Shaver, Giblin, Nadelhoffer, & Laundre, 1995) |
| 75 | ECOQUA South American Plant Traits Database – All data referencing Müller (e.g. Müller *et al.*, 2007) or Overbeck (e.g. Overbeck *et al.*, 2005) were included (criteria checked with listed references) |
| 77 | (Devictor et al., 2010) |
| 84 | Soudzilovskaia, N.A. and Onipchenko, V.G., unpublished data |
| 87 | Global leaf Robustness and Physiology Database (Niinemets, 1999) – Only data originating from the following references were included: (Bongers & Pompa, 1990; Morales, Gonzalez-Rodriguez, & Cermak, 1996) |
| 88 | (Ordoñez et al., 2010) |
| 91 | (Ogaya & Peñuelas, 2003) |
| 94 | Global A, N, P, SLA Database (Reich, Oleksyn, & Wright, 2009) – Only data originating from the following references were included: (D. Ackerly, 2004; Baddeley, Woodin, & Alexander, 1994) |
| 100 | Anand, M., unpublished data |
| 102 | Blonder, B., unpublished data |
| 105 | (Freschet, Cornelissen, van Logtestijn, & Aerts, 2010) |
| 112 | (S. J. Wright et al., 2010) |
| 113 | (Messier, McGill, & Lechowicz, 2010) |
| 114 | (Penuelas et al., 2010) |
| 115 | Hickler, T., unpublished data |
| 123 | VirtualForests Trait Database (Gutiérrez & Huth, 2012) – Only data originating from the following references were included: (Gutiérrez, Armesto, & Aravena, 2004; Parada, Jara, & Lusk, 2003) |
| 131 | Penuelas, J., unpublished data |
| 152 | (Adler, Milchunas, Lauenroth, Sala, & Burke, 2004) |
| 156 | (Bond-Lamberty, Wang, & Gower, 2002; Bond-Lamberty, Wang, Gower, & Norman, 2002) |
| 159 | (Butterfield & Briggs, 2011) |
| 161 | (Campetella et al., 2011) |
| 163 | (Craine & Towne, 2010; Craine, Towne, Ocheltree, & Nippert, 2012) |
| 165 | (Brown et al., 2013) |
| 170 | (Frenette-Dussault, Shipley, Léger, Meziane, & Hingrat, 2012) |
| 180 | Leaf Ash Content in Chinea’s Terrestrial Plants (W. Han et al., 2012) – All data from this database was included (criteria checked with and verified by database custodian) |
| 181 | (Chen, Han, Tang, Tang, & Fang, 2013) |
| 183 | Hattingh, W.N., unpublished data |
| 190 | (Kraft et al., 2008) |
| 193 | (Laughlin, Fulé, Huffman, Crouse, & Laliberté, 2011; Laughlin, Leppert, Moore, & Sieg, 2010) |
| 194 | (de Araujo et al., 2012) |
| 200 | (Milla & Reich, 2011) |
| 205 | (Price & Enquist, 2007) |
| 206 | (Read, Henning, Classen, & Sanders, 2018) |
| 207 | Römmermann, C., unpublished data |
| 209 | Schamp, B., unpublished data |
| 215 | Totté, A., unpublished data |
| 218 | (Holle & Simberloff, 2004) |
| 226 | (Blonder et al., 2012, 2015; Blonder, Violle, Bentley, & Enquist, 2011; Blonder, Violle, & Enquist, 2013) |
| 227 | Cerabolini, B.E.L., unpublished data |
| 228 | (Cerabolini et al., 2010) |
| 230 | (Craven et al., 2007) |
| 234 | (Kichenin, Wardle, Peltzer, Morse, & Freschet, 2013) |
| 236 | (Prentice et al., 2011) |
| 239 | (Missio et al., 2016) |
| 249 | (Sandel, Corbin, & Krupa, 2011) |
| 255 | (Figueira et al., 2008) |
| 256 | (Spasojevic & Suding, 2012) |
| 262 | (Williams, Shimabokuro, & Rastetter, 2012) |
| 263 | (Powers & Tiffin, 2010) |
| 265 | (Guy, Mischkolz, & Lamb, 2013) |
| 267 | Gonzalez-Melo, A., unpublishd data |
| 269 | (Baraloto et al., 2010) |
| 270 | (Peco, De Pablos, Traba, & Levassor, 2005) |
| 274 | Lenti, F.E.B., unpublished data |
| 329 | (Soudzilovskaia et al., 2013) |

**Table S1.2** List of references of studies from the TTT database that are included in this study. We show the dataset names as given in TTT and the original references per dataset of the data that was included in our database. In the case of unpublished data, the criteria were checked with the dataset custodians. The full references of the original data sources can be found in Appendix S3.

| **Dataset name** | **References** |
| --- | --- |
| **Alba Anadon-Rosell, Josep M Ninot** | Unpublished data |
| **Anne Bjorkman, Greg Henry** | (Bjorkman, Elmendorf, Beamish, Vellend, & Henry, 2015) |
| **Catherine Pickering** | (Pickering & Venn, 2013) |
| **Chelsea Little, Juha Alatalo, Ulf Molau** | Unpublished data |
| **Chelsea Little, Elisabeth Cooper** | (Little, Cutting, Alatalo, & Cooper, 2017) |
| **Daan Blok** | (Nauta et al., 2015) |
| **Elina Kaarlejarvi; Anu Eskelinen; Johan Olofsson** | (Kaarlejärvi, Eskelinen, & Olofsson, 2017) |
| **Elina Kaarlejarvi** | (Kaarlejärvi et al., 2012) |
| **Elina Kaarlejarvi, Anu Eskelinen, Johan Olofsson** | (Eskelinen, Kaarlejärvi, & Olofsson, 2017) |
| **Esther Frei, Greg Henry** | Unpublished data |
| **Greg Henry** | Unpublished data |
| **Iturrate-Garcia, Gabriela Schaepman-strub** | (Iturrate-Garcia et al., 2016) |
| **James David Mervyn Speed** | (Speed, Austrheim, Hester, & Mysterud, 2010) |
| **James Hudson, Greg Henry** | (Hudson, Henry, & Cornwell, 2011) |
| **Josep Maria Ninot, Oriol Grau** | (Grau, Ninot, Pérez-Haase, & Callaghan, 2014) |
| **Karen Harper** | (Harper, Lavallee, & Dodonov, 2018) |
| **Logan Berner, Heather D Alexander** | (Berner et al., 2015) |
| **Lorna Street** | (Street et al., 2018) |
| **Mariska Te Beest** | Unpublished data |
| **Martin Moersdorf, Elisabeth Cooper** | (Mörsdorf et al., n.d.) |
| **Megan Good; Peter Green** | (Good, Morgan, Venn, & Green, 2018) |
| **Megan Good; Peter Green; John Morgan** | Unpublished data |
| **Michele Carbognani** | (Carbognani, Petraglia, & Tomaselli, 2014) |
| **Philipp Semenchuck, Sabine Rumpf, Elizabeth Cooper, Bo Elberling** | (Rumpf, Semenchuk, Dullinger, & Cooper, 2014) |
| **Rebecca Klady, Greg Henry** | (Klady, Henry, & Lemay, 2011) |
| **Robert Hollister** | (Barrett, Hollister, Oberbauer, & Tweedie, 2015) |
| **Signe Normand, Urs Treier** | Unpublished data |
| **Susanna Venn, Sonja Wipf** | Unpublished data |
| **Tage Vowles, Robert Bjork** | (Vowles et al., 2017) |

**Appendix S2.** Data selection and cleaning

We requested data from the TRY Plant Trait Database in November 2016 and received 164 datasets. After assessing geographical coverage of these datasets, we contacted data contributors of specific datasets covering areas where we lacked data, adding 4 extra datasets. In total, we obtained 168 datasets, comprising 964,464 trait records.

Further, we collated data from the Tundra Trait Team (TTT) database (Bjorkman et al., 2018). The TTT database includes data from 66 datasets and comprises of 41,255 trait records.

Since all datasets come from disparate sources with different quality, location uncertainty, origin and representativeness, we established a set of stringent criteria to select plant trait data that was comparatively of high quality for inclusion in our analysis (Table S2). From the TRY database, we deleted 63.1% of the datasets completely, and additionally we deleted specific observations of 34.6% of the remaining datasets. From the TTT database, we deleted 54.5% of all datasets.

We checked and corrected for possible errors in our database by doing the following: 1) we removed duplicate entries, defined by having the same coordinates and references or the same coordinates and trait values but different dataset identification numbers; 2) for observations not located on land, we checked the original reference and corrected coordinates according to the site description; 3) we excluded obvious errors in trait observations (e.g. SLA = 0); and 4) we evaluated outliers in trait values against values of the same species reported by other studies in our database and/or external sources (local floras), and corrected them when appropriate (adjustment of decimal separator).

**Table S2** List of data selection criteria and their explanation. We included the number of complete datasets that were deleted per criterion (*Complete*) and the number of datasets where part of the trait records were deleted (*Part*) after checking the references from the original data or after querying the data contributors.

| Criterion | Explanation |  | *TRY* | | |  | *TTT* |
| --- | --- | --- | --- | --- | --- | --- | --- |
|  |  |  | *Complete* |  | *Part* |  | *Complete* |
| Georeferenced | Longitude and Latitude should be provided. The precision was not of concern. |  | 37 |  | 4 |  | - |
| Measured | Trait data was sampled from individuals in the field. Data from flora and large-scale species trait averages were excluded. |  | 5 |  | 1 |  | - |
| Natural vegetation | Samples should be taken in natural vegetation, e.g. data from samples taken in plantations, parks, and agricultural or livestock land, as well as experimental data, were excluded. Disturbances and management were noted but not considered in the analyses. |  | 14 |  | 6 |  | - |
| Terrestrial vegetation | All data on aquatic and wetland species was excluded. |  | 2 |  | 2 |  | - |
| Representative vegetation | Samples should be taken from all species present in the community or from species that make up the dominant vegetation structure. We also included studies that sampled only the most abundant or most common species. Studies that sampled only seedlings or saplings were excluded, too. |  | 11 |  | 9 |  | 12 |
| Sampling year | Data collection needed to be done in or after 1980. |  | 1 |  | 2 |  | - |
| Traceable data | Observations needed to be referenced, so that each data point could be checked for each of the criteria. When this was not the case, the dataset custodian was contacted to clarify the data. If still not all criteria could be checked, the data was deleted. |  | 21 |  | 10 |  | 19 |
| No duplicates | Some datasets included duplicate data (from different years) or different datasets provided data on the same community (same location and trait values). In these cases, trait values were averaged or deleted. |  | 3 |  | 2 |  | 4 |
| Usefull plant traits | At least one of the selected traits should be present in each observation. This criterion also excluded datasets that contain only categorical data. |  | 8 |  | 1 |  | 1 |

**Appendix S3.** Community means

Laughlin et al. (2018) pointed out that using community means should only be considered for predictive modeling when empirical support shows that an evolutionary trade-off between traits occurred, which affected plant vital rates. This ratifies the use of community mean in global trait models, which focus on large environmental gradients, particularly when the modelled plant traits affect plant fitness. The question whether this community mean should be weighted by species abundance or not, is a different question. To the best of our knowledge, this question has never been researched at the continental or global scale, but different studies at different scales show different results on the question whether to use weighted vs. non-weighted community means. Some small scale studies show that this difference is not significant for local communities when considering plant traits (e.g. specific leaf area, wood density, height, LNC) (D. D. Ackerly & Cornwell, 2007; Douma, Shipley, Witte, Aerts, & Bodegom, 2012; Ordoñez et al., 2010). Moving to the regional scale, Borgy et al. (2017) show that considering species abundances does make a difference when looking at plant traits (SLA and LNC) and that using community weighted means has the strongest effect on detection of trait-climate relationships. However, Butler et al. (2017) compared unweighted community mean data (SLA and LNC) for forest plots in the amazon with the data from Ter Steege et al. (2013) on hyperdominance (i.e. the phenomenon of common species having large geographic ranges while only being dominant in a few regions). They concluded that equal weights (species means without weighting by abundance) produced trait distribution estimates closest to those of the hyperdominant trait abundances. This suggests that using community unweighted means would give similar results at the regional level than when community weighted means would be used.

As previous studies do not provide a conclusive answer, we chose to use the unweighted community mean out of practical reasons: this resulted in 65% more data points than when we would consider species abundance as not all studies reported this information. Additionally, the mechanism being studied (weighted vs. unweighted community means) constrains the results of the analyses, making it of lesser importance to expect a priori ‘best’ option, as long as we clearly state what mechanism we considered in the study.

**Appendix S4.** Data distribution

**Figure S4.** Locations of trait observations (black dots).

**Appendix S5.** Considered environmental predictors

We combined the full bioclimatic variable list and soil variables available in SoilGrids250m (Hengl et al., 2017). General predictors that (we expect) cannot act as a filter via affecting plant fitness via traits were directly removed, e.g. mean annual precipitation and mean annual temperature. From the soil variable list, we directly removed categorical variables and variables that were indicated to be of low accuracy (Hengl et al., 2017). Table S5 gives this overview. In conclusion, all variables that could potentially limit or filter plant traits (extremes) but had high accuracy in global predictions, where initially considered.

With the remaining variables, we ran correlation tests and based on assumed biological relevance (i.e. hypothesized filtering effect on traits) we included variables and removed others.

**Table S5** Complete list of soil and bioclimatic variables. The strikethrough variables were not considered for our analyses.

| Soil variables | Bioclimatic variables |
| --- | --- |
| Soil organic carbon content | ~~Annual Mean Temperature~~ |
| Soil pH | Mean Diurnal Range |
| Sand, silt and clay | Isothermality |
| Bulk density | Temperature Seasonality |
| Cation-exchange capacity | Max Temperature of Warmest Month |
| ~~Coarse fragments~~ | Min Temperature of Coldest Month |
| Depth to bedrock | Temperature Annual Range |
| ~~World Reference Base (WRB) classes~~ | Mean Temperature of Wettest Quarter |
| ~~USDASoil Taxonomy suborders~~ | Mean Temperature of Driest Quarter |
|  | Mean Temperature of Warmest Quarter |
|  | Mean Temperature of Coldest Quarter |
|  | ~~Annual Precipitation~~ |
|  | Precipitation of Wettest Month  Precipitation of Driest Month  Precipitation Seasonality  Precipitation of Wettest Quarter  Precipitation of Driest Quarter  Precipitation of Warmest Quarter |
|  | Precipitation of Coldest Quarter  Aridity Index |

**Appendix S6.** Model parameterization

We fitted the GLMs using both linear and quadratic terms for each of the predictors using a LASSO penalty to control for overfitting (Goeman, 2010). The LASSO applies a penalty to the coefficients so that they are shrunk towards zero to maximize predictive ability. The amount of penalization is tuned by the lambda parameter. We selected the lambda parameter that maximizes model predictive performance while minimizing over fitting. The lambda parameter selection was performed with a split-sample validation (80% training data and 20% test data, with 10 replicates), where we varied lambda from 0 to 2000 with intervals of 1. The lambda value used in the model with the highest predictive performance (based on lowest root mean square error (RMSE)) was selected to fit the full model on. This procedure of parameter selection was also used for the models described below.

We fitted GAMs using penalized regression smoothers to estimate the smooth term for each of the predictors. We used the parameter k to control for the maximum degrees of freedom allowed for each model term. Per model, we varied k from 1 to 20 (except for wood density, where k ranged from 1 to 9, as the amount of data points did not allow for more degrees of freedom in the model). As GAMs tend to over-fit even when the degrees of freedom are optimized with internal calculations (Wood, 2006), we also included the additional shrinkage term ‘gamma’, which inflates the model’s degrees of freedom. We use a gamma value of 1.4, as it has been suggested as a good compromise for reducing overfitting while not reducing model fit (Wood, 2006). We also included a double penalty to variable coefficients in the GAMs: one to reduce the amount of smoothing, and one to completely remove terms from the model (Marra & Wood, 2011).

We fitted random forest (RF) algorithms using 10,000 trees, ensuring good convergence. The number of predictors that are randomly sampled at each split of the trees was varied from 1 to 7. Finally, we fitted Boosted Regression Trees (BRTs), which mainly differ from RF in the way that single trees are combined. BRT uses boosting, which is known to improve predictive performance (Elith, Leathwick, & Hastie, 2008). Five parameters need to be set in order to run BRT: learning rate, interaction depth, bag fraction, the number of trees grown, and the minimum number of observations in a node. We grew 3000 trees, and set the minimum number of observations in a node to 10. For the parameter selection, we systematically varied the learning rate taking the value 0.001, 0.01, 0.05 or 0.1, the interaction depth from 1 to 7, and the bag fraction taking the value 0.5, 0.6, or 0.7.

The response variables SLA, LNC, and plant height were log-transformed to improve model performance. For the GLM and GAM, we also log-transformed the humidity index, CEC, precipitation seasonality, precipitation in the driest quarter, and fire frequency. For the GLMs, we scaled all predictors in order to ensure an unbiased effect of the predictor selection.

All analyses were conducted in R 3.3.2 (R Core Team, 2016) using the ‘penalized’ package (Goeman, 2010) for the GLMs, the ‘mgcv’ package (Wood, 2004) for the GAMs, the ‘RandomForest’ package (Liaw & Wiener, 2002) for RFs, and the ‘gbm’ package (Ridgeway, 2017) for the BRTs. The script for model parameter selection is included in the Supplementary Material.

**Appendix S7.** Assessing the effect of including intra-specific trait variation and species representativeness

To test the effect of data quality on the predictability of traits, we created our different datasets with trait values for the same communities.

Following Figure S7, the datasets including intra-specific trait variation include data that is selected based on the selection criteria in Table S2. The datasets including inter-specific trait values were calculated by averaging trait values per species of all the data we originally gathered, and replacing the original trait values with these species averages. Besides the individually collected datasets, this includes the entire database of the Tundra Trait Team (TTT), and the following DatasetIDs from TRY: 49, 1, 37, 10, 130 21, 50, 51, 25, 54, 53, 52, 27, 55, 48, 87, 3, 34, 57, 4, 60, 61, 65, 63, 66, 56, 68, 70, 35, 72, 73, 75, 79, 80, 12, 92, 88, 91, 94, 95, 77, 96, 97, 98, 113, 105, 102, 129, 115, 116, 114, 104, 106, 110, 119, 112, 100, 101, 122, 125, 133, 108, 20,, 64,, 74,, 45,, 67,, 278, 150, 156, 158, 159, 160, 161, 163, 164, 166, 167, 168, 169, 170, 171, 172, 175, 176, 178, 179, 180, 186, 183, 184, 187, 188, 190, 152, 192, 193, 194, 196, 197, 199, 202, 203, 204, 205, 206, 207, 209, 210, 211, 212, 200, 181, 189, 173, 154, 191, 174, 215, 216, 217, 218, 220, 221, 223, 226, 227, 228, 229, 255, 234, 236, 237, 238, 240, 243, 248, 252, 241, 231, 246, 247, 230, 249, 123, 256, 263, 262, 264, 265, 266, 267, 277, 131, 251, 273, 269, 270, 274, 271, 275, 239, 279, 280. To calculate the global species trait averages we had 8484 species. On average, we had 703.2 trait values per species to average over, with a median of 127 trait values, a minimum of 1 trait value, and a maximum of 10270 trait values.

Following Figure S7, the datasets including representative data include data that is selected based on the selection criteria in Table S2. Non-representative datasets, where species representativeness of the entire plant community was not considered, were simulated by randomly selecting one species from each community, replicating this process 10 times for both the inter- and intra-specific trait variation dataset. This one-species sample mimicks common practice where data from the Global Biodiversity Information Facility (GBIF) are matched with independently collected trait values.

For each dataset, community means were then calculated as described in section 2.3 of the main text. This most likely gives an underestimation of the impact of species representativeness on the predictability of traits since the single species selected from the community is drawn from an already representative sample of species instead of the entire plant community.

To ensure pure effects of trait values and species representativeness, and not of sample size effects or differences in applicability domain (i.e. the area where model predictions are assumed correct due to the environmental similarities), we only adjusted communities that are present in the dataset with the actual community means.

For each of the 22 datasets and per trait we ran the model parameterization (ten-fold cross-validation) and the final four models. We changed the range over which parameters could vary in the model parameter selection, because we saw with the complete parameter selection from Appendix S6 that values never reached the extremes we set. In the parameter selection for these data sets, the lambda parameter for the general linear models could only range from 0 to 10 instead of from 0 to 20. For the general additive model, we varied k from 1 to 15 instead of 1 to 20. To calculate the models’ predictive performance (cross-validated pseudo-R^2^) we regressed the predicted values and actual community means as observed trait values (i.e. the cleaned data used in the rest of the paper) for each repetition and averaged these R^2^ values to get one value for the cross-validation. We are aware that these changes may influence the final parameter settings, but since these results are only used to compare amongst each other and not for the further analysis in this paper, we decided that this is the better compromise. For the datasets with non-representative species, the ensemble cross-validated pseudo-R^2^ of each dataset was averaged at the end to obtain a single ensemble cross-validated pseudo-R^2^ value for those datasets.

**Figure S7.** Overview of data quality types. Each plot presents the same plant community, where the individuals selected to sample are displayed in green. These samples are merely an example of how samples can be representative or not representative (as defined in Methods section 2.2, criterion 4). Solid lines represent locally measured trait values, and dashed lines represent global species mean trait values. In plot 1, all adult individuals of all species of the dominant vegetation structure are selected for sampling, thus a representative sample of the community, and intra-specific trait variation is included. In plot 3, the same representative individuals are sampled as in plot 1, but intra-specific trait variation is excluded. In plot 2, only the saplings of one species of the dominant vegetation structure are sampled, constituting an unrepresentative sample of the community, yet intra-specific trait variation is included. In plot 4, the same unrepresentative individuals are sampled as in plot 3, but intra-specific trait variation is excluded.

**Table S7** Predictive performances (ten-fold cross-validated R^2^ from 80-20 split-sample approach) for four models and the ensemble for four traits using community means calculated with different data quality. The datasets are indicated by the type of data it includes. Inter = inter specific trait values. Intra = intra specific trait values. R = species that are representative of the complete plant community. NR = species that are not representative of the complete plant community.

| Trait | model | Intra  R | Intra  NR | Inter  R | Inter NR |
| --- | --- | --- | --- | --- | --- |
| **SLA** | GLM | 0.19 | 0.18 | 0.15 | 0.18 |
|  | GAM | 0.21 | 0.21 | 0.20 | 0.20 |
|  | RF | 0.31 | 0.29 | 0.41 | 0.29 |
|  | BRT | 0.23 | 0.19 | 0.27 | 0.18 |
| **LNC** | GLM | 0.00 | 0.01 | 0.00 | 0.00 |
|  | GAM | 0.00 | 0.00 | 0.02 | 0.01 |
|  | RF | 0.25 | 0.18 | 0.18 | 0.13 |
|  | BRT | 0.21 | 0.11 | 0.13 | 0.08 |
| **Height** | GLM | 0.67 | 0.61 | 0.57 | 0.56 |
|  | GAM | 0.60 | 0.56 | 0.46 | 0.39 |
|  | RF | 0.55 | 0.53 | 0.56 | 0.57 |
|  | BRT | 0.48 | 0.44 | 0.52 | 0.46 |
| **Wood** | GLM | 0.27 | 0.19 | 0.11 | 0.15 |
| **density** | GAM | 0.25 | 0.20 | 0.19 | 0.17 |
|  | RF | 0.38 | 0.31 | 0.32 | 0.24 |
|  | BRT | 0.36 | 0.21 | 0.38 | 0.19 |

**Appendix S8.** Coefficient of variation of trait predictions

For each trait (*t*), we calculated the coefficient of variation (*CV*) weighted by model performance. We calculated the standard deviation in model predictions as the difference between each model’s (*m*) prediction (*pred*) and the ensemble prediction (*obs*) (Equation 1). Then, we weighted the standard deviation by the models predictive performance (cross-validated pseudo-R^2^ values from Appendix S7, Table S7)(*r^2^*).

$${CV}_{t}=\frac{\frac{\sqrt{{\sum_{m=1}^{4} ({{pred}_{m,t}-{obs}_{t})}^{2}} * r_{m,t}^{2}}}{\sum_{m=1}^{4} r_{m,t}^{2}}}{{obs}_{t}} (1)$$

**Appendix S9.** Unrealistic predicted trait combinations

**Figure S9** Grid cells where predicted trait combinations are not observed in our own database.

**Appendix S10.** Environmental coverage of trait data

**Figure S10** Environmental coverage per trait for all predictors. The grey violin plots show the global environmental distribution and range per predictor. The coloured violin plots show the distribution and range of the trait data over the environmental coverage for a) SLA, b) LNC, c) height, d) and wood density. Predictors are minimum temperature (Tmin; °C), humidity index (HumInd; unitless), precipitation in the driest quarter of the year (PrecDryQ; mm), precipitation seasonality (PrecSeas; %), soil cation exchange capacity (CEC; cmol+ kg^-1^), and soil pH (-log(H^+^)).

**Appendix S11.** Global environmental variation

**Figure S11** Global distribution (left column) and variation (right column) of all environmental predictors. The global distribution is the average value of grid cells at the 50 by 50 kilometer resolution, and global the variation is the range (maximum value – minimum value) within each grid cell compared to the global range (maximum value – minimum value) expressed as a %. Predictors are minimum temperature (Tmin) with a maximum variation of 49.6%, humidity index (HumInd) with a maximum variation of 41.5%, precipitation in the driest quarter of the year (PrecDryQ) with a maximum variation of 62.4%, precipitation seasonality (PrecSeas) with a maximum variation of 70.9%, soil cation exchange capacity (CEC) with a maximum variation of 108.7%, and soil pH with a maximum variation of 81.0%.

**References**

Ackerly, D. (2004). Functional strategies of chaparral shrubs in relation to seasonal water deficit and disturbance. *Ecological Monographs*, *74*(1), 25–44.

Ackerly, D. D., & Cornwell, W. K. (2007). A trait-based approach to community assembly: Partitioning of species trait values into within- and among-community components. *Ecology Letters*, *10*, 135–145. http://doi.org/10.1111/j.1461-0248.2006.01006.x

Adler, P. B., Milchunas, D. G., Lauenroth, W. K., Sala, O. E., & Burke, I. C. (2004). Functional traits of graminoids in semi-arid steppes: A test of grazing histories. *Journal of Applied Ecology*, *41*, 653–663.

Baddeley, J. A., Woodin, S. J., & Alexander, I. J. (1994). Effects of increased Nitrogen and Phosphorus availability on the photosynthesis and nutrient relations of three Arctic dwarf Shrubs from Svalbard. *Functional Ecology*, *8*, 676–685.

Baraloto, C., Paine, C. E. T., Poorter, L., Beauchene, J., Bonal, D., Domenach, A. M., … Chave, J. (2010). Decoupled leaf and stem economics in rain forest trees. *Ecology Letters*, *13*, 1338–1347. http://doi.org/10.1111/j.1461-0248.2010.01517.x

Barrett, R. T. S., Hollister, R. D., Oberbauer, S. F., & Tweedie, C. E. (2015). Arctic plant responses to changing abiotic factors in northern Alaska. *American Journal of Botany*, *102*(12), 2020–2031.

Baruch, Z., & Goldstein, G. (1999). Leaf construction cost, nutrient concentration, and net CO2 assimilation of native and invasive species in Hawaii. *Oecologia*, *121*, 183–192.

Bassow, S. L., & Bazzaz, F. A. (1997). Intra- and inter-specific variation in canopy photosynthesis in a mixed deciduous forest. *Oecologia*, *109*, 507–515.

Berner, L. T., Alexander, H. D., Loranty, M. M., Ganzlin, P., Mack, M. C., Davydov, S. P., & Goetz, S. J. (2015). Biomass allometry for alder, dwarf birch, and willow in boreal forest and tundra ecosystems of far northeastern Siberia and north-central Alaska. *Forest Ecology and Management*, *337*, 110–118. http://doi.org/10.1016/j.foreco.2014.10.027

Bjorkman, A. D., Elmendorf, S. C., Beamish, A. L., Vellend, M., & Henry, G. H. R. (2015). Contrasting effects of warming and increased snowfall on Arctic tundra plant phenology over the past two decades. *Global Change Biology*, *21*, 4651–4661.

Bjorkman, A. D., Myers-Smith, I. H., Elmendorf, S. C., Normand, S., Rüger, N., Beck, P. S. A., … Weiher, E. (2018). Plant functional trait change across a warming tundra biome. *Nature*, *562*, 57–62. http://doi.org/10.1038/s41586-018-0563-7

Blonder, B., Buzzard, V., Simova, I., Sloat, L., Boyle, B., Lipson, R., … Enquist, B. J. (2012). The leaf-area shrinkage effect can bias paleoclimate and ecology research. *American Journal of Botany*, *99*(11), 1756–1763.

Blonder, B., Vasseur, F., Violle, C., Shipley, B., Enquist, B. J., & Vile, D. (2015). Testing models for the leaf economics spectrum with leaf and whole-plant traits in Arabidopsis thaliana. *AoB PLANTS*, *7*, plv049.

Blonder, B., Violle, C., Bentley, L. P., & Enquist, B. J. (2011). Venation networks and the origin of the leaf economics spectrum. *Ecology Letters*, *14*, 91–100. http://doi.org/10.1111/j.1461-0248.2010.01554.x

Blonder, B., Violle, C., & Enquist, B. J. (2013). Assessing the causes and scales of the leaf economics spectrum using venation networks in Populus tremuloides. *Journal of Ecology*, *101*, 981–989.

Bloomfield, K. J., Cernusak, L. A., Eamus, D., Ellsworth, D. S., Prentice, I. C., Wright, I. J., … Atkin, O. K. (2018). A continental-scale assessment of variability in leaf traits: within species, across sites and between seasons. *Functional Ecology*, 1–15. http://doi.org/10.1111/1365-2435.13097

Bond-Lamberty, B., Wang, C., & Gower, S. T. (2002). Aboveground and belowground biomass and sapwood area allometric equations for six boreal tree species of northern Manitoba. *Canadian Journal of Forest Research*, *32*(8), 1441–1450.

Bond-Lamberty, B., Wang, C., Gower, S. T., & Norman, J. (2002). Leaf area dynamics of a boreal black spruce fire chronosequence. *Tree Physiology*, *22*(14), 993–1001.

Bongers, F., & Pompa, J. (1990). Leaf characteristics of the tropical rain forest flora of Los Tuxtlas, Mexico. *Botanical Gazette*, *151*(3), 354–365.

Borgy, B., Violle, C., Choler, P., Denelle, P., Munoz, F., Kattge, J., … Garnier, E. (2017). Plant community structure and nitrogen inputs modulate the climate signal on leaf traits. *Global Ecology and Biogeography*, *26*(10), 1138–1152. http://doi.org/10.1111/geb.12623

Brown, K. A., Johnson, S. E., Parks, K. E., Holmes, S. M., Ivoandry, T., Abram, N. K., … Wright, P. C. (2013). Use of provisioning ecosystem services drives loss of functional traits across land use intensification gradients in tropical forests in Madagascar. *Biological Conservation*, *161*, 118–127.

Butler, E. E., Datta, A., Flores-moreno, H., Chen, M., Wythers, K. R., Fazayeli, F., … Reich, P. B. (2017). Mapping local and global variability in plant trait distributions. *Proceedings of the National Academy of Sciences*, *114*, E10937–E10946. http://doi.org/10.1073/pnas.1708984114

Butterfield, B. J., & Briggs, J. M. (2011). Regeneration niche differentiates functional strategies of desert woody plant species. *Oecologia*, *165*, 477–487.

Campetella, G., Botta-Dukát, Z., Wellstein, C., Canullo, R., Gatto, S., Chelli, S., … Bartha, S. (2011). Patterns of plant trait-environment relationships along a forest succession chronosequence. *Agriculture, Ecosystems and Environment*, *145*(1), 38–48.

Carbognani, M., Petraglia, A., & Tomaselli, M. (2014). Warming effects and plant trait control on the early-decomposition in alpine snowbeds. *Plant and Soil*, *376*(1), 277–290.

Cerabolini, B. E. L., Brusa, G., Ceriani, R. M., de Andreis, R., Luzzaro, A., & Pierce, S. (2010). Can CSR classification be generally applied outside Britain? *Plant Ecology*, *210*(2), 253–261.

Chapin, F. S. I., & Shaver, G. R. (1985). Individualistic growth response of tundra plant species to environmental manipulations in the field. *Ecology*, *66*(2), 564–576.

Chapin, F. S. I., Shaver, G. R., Giblin, A. E., Nadelhoffer, K. J., & Laundre, J. A. (1995). Responses of Arctic tundra to experimental and observed changes in climate. *Ecology*, *76*(3), 694–711.

Chen, Y., Han, W., Tang, L., Tang, Z., & Fang, J. (2013). Leaf nitrogen and phosphorus concentrations of woody plants differ in responses to climate, soil and plant growth form. *Ecography*, *36*(2), 178–184.

Cornelissen, A. J. H. C., Diez, P. C., & Hunt, R. (1996). Seedling growth, allocation and leaf attributes in a wide range of woody plant species and types. *Journal of Ecology*, *84*(5), 755–765.

Cornelissen, J. H. C., Cerabolini, B., Castro-Díez, P., Villar-Salvador, P., Montserrat-Martí, G., Puyravaud, J. P., … Aerts, R. (2003). Functional traits of woody plants: Correspondence of species rankings between field adults and laboratory-grown seedlings? *Journal of Vegetation Science*, *14*(3), 311–322. http://doi.org/10.1111/j.1654-1103.2003.tb02157.x

Cornelissen, J. H. C., Quested, H. M., Gwynn-Jones, D., Van Logtestijn, R. S. P., De Beus, M. A. H., Kondratchuk, A., … Aerts, R. (2004). Leaf digestibility and litter decomposability are related in a wide range of subarctic plant species and types. *Functional Ecology*, *18*, 779–786.

Cornwell, W. K., & Ackerly, D. D. (2009). Community assembly and shifts in plant trait distributions across an environmental gradient in coastal California. *Ecological Monographs*, *79*(1), 109–126.

Craine, J. M., & Towne, E. G. (2010). High leaf tissue density grassland species consistently more abundant across topographic and disturbance contrasts in a North American tallgrass prairie. *Plant and Soil*, *337*, 193–203.

Craine, J. M., Towne, E. G., Ocheltree, T. W., & Nippert, J. B. (2012). Community traitscape of foliar nitrogen isotopes reveals N availability patterns in a tallgrass prairie. *Plant and Soil*, *356*, 395–403.

Craven, D., Braden, D., Ashton, M. S., Berlyn, G. P., Wishnie, M., & Dent, D. (2007). Between and within-site comparisons of structural and physiological characteristics and foliar nutrient content of 14 tree species at a wet, fertile site and a dry, infertile site in Panama. *Forest Ecology and Management*, *238*, 335–346.

de Araujo, A. C., Ometto, J. P. H. B., Dolman, A. J., Kruijt, B., Waterloo, M. J., & Ehleringer, J. R. (2012). LBA-ECO CD-02 C and N isotopes in leaves and atmospheric CO2, Amazonas, Brazil, *Data set.*, Oak Ridge National Laboratory Distributed Active A. http://doi.org/http://dx.doi.org/10.3334/ORNLDAAC/1097

Devictor, V., Mouillot, D., Meynard, C., Jiguet, F., Thuiller, W., & Mouquet, N. (2010). Spatial mismatch and congruence between taxonomic, phylogenetic and functional diversity: the need for integrative conservation strategies in a changing world. *Ecology Letters*, *13*, 1030–1040.

Diemer, M. (1998). Leaf lifespans of high-elevation, aseasonal Andean shrub species in relation to leaf traits and leaf habit. *Global Ecology and Biogeography Letters*, *7*(6), 457–465.

Diemer, M. (1998). Life span and dynamics of leaves of herbaceous perennials in high-elevation environments: ‘news from the elephant’ s leg’. *Functional Ecology*, *12*, 413–425.

Diemer, M., Körner, C., & Prock, S. (1992). Leaf life spans in wild perennial herbaceous plants: a survey and attempts at a functional interpretation. *Oecologia*, *89*, 10–16.

Douma, J. C., Shipley, B., Witte, J.-P. M., Aerts, R., & Bodegom, P. M. van. (2012). Disturbance and resource availability act differently on the same suite of plant traits: revisiting assembly hypotheses. *Ecology*, *93*(4), 825–835.

Elith, J., Leathwick, J. R., & Hastie, T. (2008). A working guide to boosted regression trees. *Journal of Animal Ecology*, *77*(4), 802–813. http://doi.org/10.1111/j.1365-2656.2008.01390.x

Ellsworth, D. S., & Reich, P. B. (1993). Canopy structure and vertical patterns of photosynthesis and related leaf traits in a deciduous forest. *Oecologia*, *96*, 169–178.

Eskelinen, A., Kaarlejärvi, E., & Olofsson, J. (2017). Herbivory and nutrient limitation protect warming tundra from lowland species’ invasion and diversity loss. *Global Change Biology*, *23*, 245–255.

Figueira, A. M. e S., Miller, S. D., de Sousa, C. A. D., Menton, M. C., Maia, A. R., da Rocha, H. R., & Goulden, M. L. (2008). Effects of selective logging on tropical forest tree growth. *Journal of Geophysical Research*, *113*, G00B05. http://doi.org/10.1029/2007JG000577

Frenette-Dussault, C., Shipley, B., Léger, J. F., Meziane, D., & Hingrat, Y. (2012). Functional structure of an arid steppe plant community reveals similarities with Grime’s C-S-R theory. *Journal of Vegetation Science*, *23*, 208–222.

Freschet, G. T., Cornelissen, J. H. C., van Logtestijn, R. S. P., & Aerts, R. (2010). Evidence of the “plant economics spectrum” in a subarctic flora. *Journal of Ecology*, *98*(2), 362–373.

Fyllas, N. M., Patino, S., Baker, T. R., Bielefeld Nardoto, G., Martinelli, L. A., Quesada, C. A., … Lloyd, J. (2009). Basin-wide variations in foliar properties of Amazonian forest: Phylogeny, soils and climate. *Biogeosciences*, *6*, 2677–2708.

Garnier, E., Laurent, G., Bellmann, A., Debain, S., Berthelier, P., Ducout, B., … Navas, M.-L. (2001). Consistency leaf traits species ranking based on functional leaf traits. *New Phytologist*, *152*, 69–83.

Garnier, E., Lavorel, S., Ansquer, P., Castro, H., Cruz, P., Dolezal, J., … Zarovali, M. P. (2007). Assessing the effects of land-use change on plant traits, communities and ecosystem functioning in grasslands: A standardized methodology and lessons from an application to 11 European sites. *Annals of Botany*, *99*(5), 967–985.

Gleason, S. M., Butler, D. W., Ziemińska, K., Waryszak, P., & Westoby, M. (2012). Stem xylem conductivity is key to plant water balance across Australian angiosperm species. *Functional Ecology*, *26*, 343–352. http://doi.org/10.1111/j.1365-2435.2012.01962.x

Goeman, J. J. (2010). L1 penalized estimation in the Cox proportional hazards model. *Biometrical Journal*, *52*(1), 80–84.

Good, M. K., Morgan, J. W., Venn, S., & Green, P. (2018). Timing of snowmelt affects species composition via plant strategy filtering. *Basic and Applied Ecology*, *under revi*.

Grau, O., Ninot, J. M., Pérez-Haase, A., & Callaghan, T. V. (2014). Plant co-existence patterns and high-arctic vegetation composition in three common plant communities in north-east Greenland. *Polar Research*, *33*(2014). http://doi.org/10.3402/polar.v33.19235

Gutiérrez, A. G., Armesto, J. J., & Aravena, J. C. (2004). Disturbance and regeneration dynamics of an old-growth North Patagonian rain forest in Chiloé Island, Chile. *Journal of Ecology*, *92*, 598–608.

Gutiérrez, A. G., & Huth, A. (2012). Successional stages of primary temperate rainforests of Chiloé Island, Chile. *Perspectives in Plant Ecology, Evolution and Systematics*, *14*, 243–256.

Guy, A. L., Mischkolz, J. M., & Lamb, E. G. (2013). Limited effects of simulated acidic deposition on seedling survivorship and root morphology of endemic plant taxa of the Athabasca Sand Dunes in well-watered greenhouse trials. *Botany*, *91*, 176–181.

Han, Q., Kawasaki, T., Nakano, T., & Chiba, Y. (2004). Spatial and seasonal variability of temperature responses of biochemical photosynthesis parameters and leaf nitrogen content within a Pinus densiflora crown. *Tree Physiology*, *24*, 737–744.

Han, W., Chen, Y., Zhao, F. J., Tang, L., Jiang, R., & Zhang, F. (2012). Floral, climatic and soil pH controls on leaf ash content in China’s terrestrial plants. *Global Ecology and Biogeography*, *21*, 376–382.

Harper, K. A., Lavallee, A. A., & Dodonov, P. (2018). Patterns of shrub abundance and relationships with other plant types within the forest-tundra ecotone in northern Canada. Arctic Science.

Hengl, T., Jesus, J. M. De, Heuvelink, G. B. M., Ruiperez Gonzalez, M., Kilibarda, M., Blagoti, A., … Kempen, B. (2017). SoilGrids250m: Global gridded soil information based on machine learning. PLoS ONE, 12(2). http://doi.org/10.1371/journal.pone.0169748

Hikosaka, K., & Hirose, T. (2000). Photosynthetic nitrogen-use efficiency in evergreen broad-leaved woody species coexisting in a warm-temperate forest. *Tree Physiology*, *20*, 1249–1254.

Hikosaka, K., Nagamfatsu, D., Ishii, H. S., & Hirose, T. (2002). Photosynthesis – nitrogen relationships in species at different altitudes on Mount Kinabalu, Malaysia. *Ecological Research*, *17*, 305–313.

Holle, B. Von, & Simberloff, D. (2004). Testing Fox’s assembly rule: does plant invasion depend on recipient community structure? *Oikos*, *105*, 551–563.

Hudson, J. M. G., Henry, G. H. R., & Cornwell, W. K. (2011). Taller and larger: Shifts in Arctic tundra leaf traits after 16 years of experimental warming. *Global Change Biology*, *17*, 1013–1021.

Iturrate-Garcia, M., O’Brien, M. J., Khitun, O., Abiven, S., Niklaus, P. A., & Schaepman-Strub, G. (2016). Interactive effects between plant functional types and soil factors on tundra species diversity and community composition. *Ecology and Evolution*, *6*, 8126–8137. http://doi.org/10.1002/ece3.2548

Jurik, T. W. (1986). Temporal and spatial patterns of specific leaf weight in successional northern hardwood tree species. *American Journal of Botany*, *73*(8), 1083–1092.

Jurik, T. W., Weber, J. A., & Gates, D. M. (1988). Effects of temperature and light on photosynthesis of dominant species of a northern hardwood forest. *Botanical Gazette*, *149*(2), 203–208.

Kaarlejärvi, E., Baxter, R., Hofgaard, A., Hytteborn, H., Khitun, O., Molau, U., … Olofsson, J. (2012). Effects of sarming on shrub abundance and chemistry drive ecosystem-level changes in a forest-tundra ecotone. *Ecosystems*, *15*, 1219–1233. http://doi.org/10.1007/s10021-012-9580-9

Kaarlejärvi, E., Eskelinen, A., & Olofsson, J. (2017). Herbivores rescue diversity in warming tundra by modulating trait-dependent species losses and gains. *Nature Communications*, *8*, 419. http://doi.org/10.1038/s41467-017-00554-z

Kattge, J., Knorr, W., Raddatz, T., & Wirth, C. (2009). Quantifying photosynthetic capacity and its relationship to leaf nitrogen content for global-scale terrestrial biosphere models. *Global Change Biology*, *15*, 976–991.

Kazakou, E., Vile, D., Shipley, B., Gallets, C., & Garnier, E. (2006). Co-variations in litter decomposition, leaf traits and plant growth in species from a Mediterranean old-field succession. *Functional Ecology*, *20*, 21–30.

Kichenin, E., Wardle, D. A., Peltzer, D. A., Morse, C. W., & Freschet, G. T. (2013). Contrasting effects of plant inter- and intraspecific variation on community-level trait measures along an environmental gradient. *Functional Ecology*, *27*(5), 1254–1261.

Klady, R. A., Henry, G. H. R., & Lemay, V. (2011). Changes in high arctic tundra plant reproduction in response to long-term experimental warming. *Global Change Biology*, *17*, 1611–1624.

Kraft, N. J. B., Valencia, R., & Ackerly, D. D. (2008). Functional traits and niche-based tree community assembly in an Amazonian forest. *Science*, *322*, 580–582.

Kudo, G. (1996). Intraspecific variation of leaf traits in several deciduous species in relation to length of growing season. *Écoscience*, *3*(4), 483–489.

Kudo, G., Molau, U., & Wada, N. (2001). Leaf-trait variation of tundra plants along a climatic gradient: An integration of responses in evergreen and deciduous species. *Arctic, Antarctic, and Alpine Research*, *33*(2), 181–190.

Kurokawa, H., & Nakashizuka, T. (2008). Leaf herbivory and decomposability in a Malaysian tropical rain forest. *Ecology*, *89*(9), 2645–2656.

Laughlin, D. C., Fulé, P. Z., Huffman, D. W., Crouse, J., & Laliberté, E. (2011). Climatic constraints on trait-based forest assembly. *Journal of Ecology*, *99*, 1489–1499.

Laughlin, D. C., Leppert, J. J., Moore, M. M., & Sieg, C. H. (2010). A multi-trait test of the leaf-height-seed plant strategy scheme with 133 species from a pine forest flora. *Functional Ecology*, *24*, 493–501.

Laughlin, D. C., Strahan, R. T., Adler, P. B., & Moore, M. M. (2018). Survival rates indicate that correlations between community-weighted mean traits and environments can be unreliable estimates of the adaptive value of traits. *Ecology Letters*, *21*(3), 411–421. http://doi.org/10.1111/ele.12914

Liaw, A., & Wiener, M. (2002). Classification and regression by randomForest. *R News*, *2*(3), 18–22.

Little, C. J., Cutting, H., Alatalo, J., & Cooper, E. J. (2017). Short-term herbivory has long-term consequences in warmed and ambient high Arctic tundra. *Environmental Research Letters*, *12*, 025001.

Marra, G., & Wood, S. N. (2011). Practical variable selection for generalized additive models. *Computational Statistics and Data Analysis*, *55*, 2372–2387. http://doi.org/10.1016/j.csda.2011.02.004

Mcallister, C. A., Knapp, A. K., & Maragni, L. A. (1998). Is leaf-level photosynthesis related to plant success in a highly productive grassland? *Oecologia*, *117*, 40–46.

Medlyn, B., Badeck, F.-W., De Pury, D., Barton, C., Broadmeadow, M., Ceulemans, R., … Jarvis, P. (1999). Effects of elevated [CO2] on photosynthesis in European forest species: a meta-analysis of model parameters. *Plant Cell and Environment*, *22*, 1475–1495.

Meir, P., Levy, P. E., Grace, J., & Jarvis, P. G. (2007). Photosynthetic parameters from two contrasting woody vegetation types in West Africa. *Plant Ecology*, *192*(2), 277–287. http://doi.org/10.1007/s11258-007-9320-y

Messier, J., McGill, B. J., & Lechowicz, M. J. (2010). How do traits vary across ecological scales? A case for trait-based ecology. *Ecology Letters*, *13*, 838–848.

Midgley, J. J., van Wyk, G. R., & Everard, D. A. (1995). Leaf attributes of South African forest species. *African Journal of Ecology*, *33*, 160–168.

Milla, R., & Reich, P. B. (2011). Multi-trait interactions, not phylogeny, fine-tune leaf size reduction with increasing altitude. *Annals of Botany*, *107*(3), 455–465.

Missio, F., Higuchi, P., Silva, A., Longhi, S., Salami, B., Dalla Rosa, A., … Bento, M. (2016). Trade-offs and spatial variation of functional traits of tree species in a subtropical forest in southern Brazil. *IForest*, *9*, 855–859. http://doi.org/10.3832/ifor1960-009

Mitchell, K. A., Bolstad, P. V, & Vose, J. M. (1999). Interspecific and environmentally induced variation in foliar dark respiration among eighteen southeastern deciduous tree species. *Tree Physiology*, *19*, 861–870.

Mooney, H. A., Field, C., Gulmon, S. L., & Bazzaz, F. A. (1981). Photosynthetic capacity in relation to leaf position in desert versus old-field annuals. *Oecologia*, *50*, 109–112.

Morales, D., Gonzalez-Rodriguez, A. M., & Cermak, J. (1996). Laurel forests in Tenerife, Canary Islands: The vertical profiles of leaf characteristics. *Phyton*, *36*, 251–263.

Mörsdorf, M. A., Baggesen, N. S., Yoccoz, N. G., Michelsen, A., Elberling, B., Ambus, P. L., & Cooper, E. J. (n.d.). Deep snow regimes impose higher amounts of labile soil nitrogen during the growing season, and lead to increased inorganic N uptake by high Arctic tundra plants.

Müller, S. C., Overbeck, G. E., Pfadenhauer, J., & Pillar, V. D. (2007). Plant functional types of woody species related to fire disturbance in forest-grassland ecotones. *Plant Ecology*, *189*, 1–14.

Nauta, A. L., Heijmans, M. M. P. D., Blok, D., Limpens, J., Elberling, B., Gallagher, A., … Berendse, F. (2015). Permafrost collapse after shrub removal shifts tundra ecosystem to a methane source. *Nature Climate Change*, *5*, 67–70.

Niinemets, Ü. (1999). Components of leaf dry mass per area – thickness and density – alter leaf photosynthetic capacity in reverse directions in woody plants. *New Phytologist*, *144*, 35–47.

Ogaya, R., & Peñuelas, J. (2003). Comparative field study of Quercus ilex and Phillyrea latifolia: Photosynthetic response to experimental drought conditions. *Environmental and Experimental Botany*, *50*, 137–148.

Ordoñez, J. C., van Bodegom, P. M., Witte, J.-P. M., Bartholomeus, R. P., van Hal, J. R., & Aerts, R. (2010). Plant strategies in relation to resource supply in mesic to wet environments: does theory mirror nature? *The American Naturalist*, *175*(2), 225–239.

Overbeck, G. E., Müller, S. C., Pillar, V. D., & Pfadenhauer, J. (2005). Fine-scale post-fire dynamics in southern Brazilian subtropical grassland. *Journal of Vegetation Science*, *16*(6), 655–664.

Parada, T. J., Jara, C. V., & Lusk, C. H. (2003). Distribución de alturas máximas de especies en rodales antiguos de selva Valdiviana, Parque Nacional Puyehue. *Bosque*, 63–67.

Peco, B., De Pablos, I., Traba, J., & Levassor, C. (2005). The effect of grazing abandonment on species composition and functional traits: The case of dehesa grasslands. *Basic and Applied Ecology*, *6*, 175–183.

Penuelas, J., Sardans, J., Llusià, J., Owen, S. M., Carnicer, J., Giambelluca, T. W., … Niinemets, Ü. (2010). Faster returns on “leaf economics” and different biogeochemical niche in invasive compared with native plant species. *Global Change Biology*, *16*, 2171–2185.

Pickering, C. M., & Venn, S. (2013). *Increasing the resilience of Australian alpine flora to climate change and assosiated threats: A plant functional traits approach.*

Poorter, L. (2009). Leaf traits show different relationships with shade tolerance in moist versus dry tropical forests. *New Phytologist*, *181*, 890–900.

Poorter, L., & Bongers, F. (2011). Leaf traits are good predictors of plant performance across 53 rain forest species. *Ecology*, *87*(7), 1733–1743.

Powers, J. S., & Tiffin, P. (2010). Plant functional type classifications in tropical dry forests in Costa Rica: Leaf habit versus taxonomic approaches. *Functional Ecology*, *24*, 927–936.

Prentice, I. C., Meng, T., Wang, H., Harrison, S. P., Ni, J., & Wang, G. (2011). Evidence of a universal scaling relationship for leaf CO. *New Phytologist*, *190*, 169–180.

Price, C. A., & Enquist, B. J. (2007). Scaling mass and morphology in leaves: An extension of the WBE Model. *Ecology*, *88*(5), 1132–1141.

Pyankov, V. I., Kondratchuk, A. V., & Shipley, B. (1999). Leaf structure and specific leaf mass: the alpine desert plants of the Eastern Pamirs, Tadjikistan. *New Phytologist*, *143*, 131–142.

Quested, H. M., Cornelissen, J. H. C., Press, M. C., Callaghan, T. V, Aerts, R., Trosien, F., … Jonasson, S. E. (2003). Decomposition of sub-Arctic plants with differing nitrogen economies: A functional role for hemiparasites. *Ecology*, *84*(12), 3209–3221.

R Core Team. (2016). R: A language and environment for statistical computing. R Foundation for Statistical Computing, Vienna, Austria.

Read, Q. D., Henning, J. A., Classen, A. T., & Sanders, N. J. (2018). Aboveground resilience to species loss but belowground resistance to nitrogen addition in a montane plant community. *Journal of Plant Ecology*, *11*, 351–363. http://doi.org/10.1093/jpe/rtx015 available

Reich, P. B., Ellsworth, D. S., Walters, M. B., Vose, J. M., Greshyam, C., Volin, J. C., … Reich Peter B., Ellsworth David S., Walters Michael B., Vose James M., Greshyam Charles, Volin John C., and B. W. D. (1999). Generelity of leaf trait relationships: a test across six biomes. *Ecology*, *80*(6), 1955–1969.

Reich, P. B., Oleksyn, J., & Wright, I. J. (2009). Leaf phosphorus influences the photosynthesis-nitrogen relation: A cross-biome analysis of 314 species. *Oecologia*, *160*, 207–212.

Ridgeway, G. (2017). gbm: Generalized boosted regression models. *R Package Version 2.1.3*.

Rumpf, S. B., Semenchuk, P. R., Dullinger, S., & Cooper, E. J. (2014). Idiosyncratic responses of high arctic plants to changing snow regimes. *PLoS ONE*, *9*(2), e86281. http://doi.org/10.1371/journal.pone.0086281

Sandel, B., Corbin, J. D., & Krupa, M. (2011). Using plant functional traits to guide restoration: A case study in California coastal grassland. *Ecosphere*, *2*(2), art23. http://doi.org/doi:10.1890/ES10-00175.1

Schlesinger, W. H., DeLucia, E. H., & Billings, W. D. (1989). Nutrient-use efficiency of woody plants on contrasting soils in the western Great Basin, Nevada. *Ecology*, *70*(1), 105–113.

Simpson, A. H., Richardson, S. J., & Laughlin, D. C. (2016). Soil–climate interactions explain variation in foliar, stem, root and reproductive traits across temperate forests. *Global Ecology and Biogeography*, *25*, 964–978. http://doi.org/10.1111/geb.12457

Sobrado, M. A. (1991). Cost-benefit relationships in deciduous and evergreen leaves of tropical dry forest species. *Functional Ecology*, *5*, 608–616.

Soudzilovskaia, N. A., Elumeeva, T. G., Onipchenko, V. G., Shidakov, I. I., Salpagarova, F. S., Khubiev, A. B., … Cornelissen, J. H. C. (2013). Functional traits predict relationship between plant abundance dynamic and long-term climate warming. *Proceedings of the National Academy of Sciences*, *110*, 18180–18184.

Spasojevic, M. J., & Suding, K. N. (2012). Inferring community assembly mechanisms from functional diversity patterns: The importance of multiple assembly processes. *Journal of Ecology*, *100*, 652–661.

Speed, J. D. M., Austrheim, G., Hester, A. J., & Mysterud, A. (2010). Experimental evidence for herbivore limitation of the treeline. *Ecology*, *91*(11), 3414–3420.

Street, L. E., Subke, J.-A., Baxter, R., Dinsmore, K. J., Knoblauch, C., & Wookey, P. A. (2018). Ecosystem carbon dynamics differ between tundra shrub types in the western Canadian Arctic. *Environmental Research Letters*, *in press*. http://doi.org/10.1088/1748-9326/aad363

Ter Steege, H., Pitman, N. C. A., Sabatier, D., Baraloto, C., Salomão, R. P., Guevara, J. E., … Silman, M. R. (2013). Hyperdominance in the Amazonian tree flora. *Science*, *342*(6156). http://doi.org/10.1126/science.1243092

Van Der Sande, M. T., Arets, E. J. M. M., Peña-Claros, M., De Avila, A. L., Roopsind, A., Mazzei, L., … Poorter, L. (2016). Old-growth Neotropical forests are shifting in species and trait composition. *Ecological Monographs*, *86*(2), 228–243. http://doi.org/10.1890/15-1815.1

van der Sande, M. T., Arets, E. J. M. M., Peña-Claros, M., Hoosbeek, M. R., Cáceres-Siani, Y., van der Hout, P., & Poorter, L. (2018). Soil fertility and species traits, but not diversity, drive productivity and biomass stocks in a Guyanese tropical rainforest. *Functional Ecology*, *32*, 461–474. http://doi.org/10.1111/1365-2435.12968

van der Sande, M. T., Peña-Claros, M., Ascarrunz, N., Arets, E. J. M. M., Licona, J. C., Toledo, M., & Poorter, L. (2017). Abiotic and biotic drivers of biomass change in a Neotropical forest. *Journal of Ecology*, *105*, 1223–1234. http://doi.org/10.1111/1365-2745.12756

Villar, R., & Merino, J. (2001). Comparison of leaf construction costs in woody species with differing leaf life-spans in contrasting ecosystems. *New Phytologist*, *151*, 213–226.

Vowles, T., Gunnarsson, B., Molau, U., Hickler, T., Klemedtsson, L., & Björk, R. G. (2017). Expansion of deciduous tall shrubs but not evergreen dwarf shrubs inhibited by reindeer in Scandes mountain range. *Journal of Ecology*, *105*, 1547–1561.

Whitehead, D., Walcroft, A. S., Scott, N. A., Townsend, J. A., Trotter, C. M., & Rogers, G. N. D. (2004). Characteristics of photosynthesis and stomatal conductance in the shrubland species manuka (Leptospermum scoparium) and kanuka (Kunzea ericoides) for the estimation of annual canopy carbon uptake. *Tree Physiology*, *24*, 795–804.

Williams-linera, G. (2000). Leaf demography and leaf traits of temperate-deciduous and tropical evergreen-broadleaved trees in a Mexican montane cloud forest. *Plant Ecology*, *149*, 233–244.

Williams, M., Shimabokuro, Y. E., & Rastetter, E. B. (2012). LBA-ECO CD-09 Soil and vegetation characteristics, Tapajos National Forest, Brazil, Data set. Available on-line [http://daac.ornl.gov]. http://doi.org/http://doi.org/http://dx.doi.org/10.3334/ORNLDAAC/1104

Wood, S. N. (2004). Stable and efficient multiple smoothing parameter estimation for generalized additive models. *Journal of the American Statistical Association*, *99*, 673–686.

Wood, S. N. (2006). *Generalized additive models: An introduction with R*. *CRC*. Boca Raton, Florida: Chapman & Hall/CRC Texts in Statistical Science. http://doi.org/10.1111/j.1541-0420.2007.00905_3.x

Wright, I. A. N. J., Westoby, M., & Reich, P. B. (2002). Convergence towards higher leaf mass per area in dry and nutrient-poor habitats has different consequences for leaf life span. *Journal of Ecology*, *90*, 534–543.

Wright, I. J., Reich, P. B., & Westoby, M. (2001). Strategy shifts in leaf physiology, structure and nutrient content between species of high- and low-rainfall and high- and low-nutrient habitats. *Functional Ecology*, *15*, 423–434.

Wright, I. J., Reich, P. B., Westoby, M., Ackerly, D. D., Baruch, Z., Bongers, F., … Gulias, J. (2004). The worldwide leaf economics spectrum. *Nature*, *428*, 821–827.

Wright, S. J., Kitajima, K., Kraft, N. J. B., Reich, P. B., Wright, I. J., Bunker, D. E., … Zanne, A. E. (2010). Functional traits and the growth — mortality trade-off in tropical trees. *Ecology*, *91*(12), 3664–3674.
